# Publication bias in simulation model studies: The case of ethanol literature

**DOI:** 10.1371/journal.pone.0284715

**Published:** 2023-05-04

**Authors:** Wyatt Thompson, Hoa Hoang, Jarrett Whistance, Robert Johansson

**Affiliations:** 1 Division of Applied Social Sciences, University of Missouri-Columbia, Missouri, Columbia, United States of America; 2 American Sugar Alliance, Arlington, Virginia, United States of America; University of Benghazi, LIBYA

## Abstract

In this study, we explore the potential for publication bias using market simulation results that estimate the effect of US ethanol expansion on corn prices. We provide a new test of whether the publication process routes market simulation results into one of the following two narratives: food-versus-fuel or greenhouse gas (GHG) emissions. Our research question is whether model results with either high price or large land impact are favored for publication in one body of literature or the other. In other words, a model that generates larger price effects might be more readily published in the food-versus-fuel literature while a model that generates larger land use change and GHG emissions might find a home in the GHG emission literature. We develop a test for publication bias based on matching narrative and normalized price effects from simulated market models. As such, our approach differs from past studies of publication bias that typically focus on statistically estimated parameters. This focus could have broad implications: if in the future more studies assess publication bias of quantitative results that are not statistically estimated parameters, then important inferences about publication bias could be drawn. More specifically, such a body of literature could explore the potential that practices common in either statistical methods or other methods tend to encourage or deter publication bias. Turning back to the present case, our findings in this study do not detect a relationship between food-versus-fuel or GHG narrative orientation and corn price effects. The results are relevant to debates about biofuel impacts and our approach can inform the publication bias literature more generally.

## 1. Introduction

Biased publication causes our understandings to be wrong and can mislead current and future research efforts. Policy decisions based on science can also be misdirected by publication bias, with serious consequences for budgets and human well-being. Public perception of scientists might also be in peril [1]. “Climategate”, a scandal in which leaked emails revealed personal views of scientists working at a widely cited laboratory, had a negative effect on public trust in science [2]. Publication bias, or publication selection, has been defined as the process of favoring research results for statistical significance [3] and can be caused by authors, editors, or reviewers [4]. Authors might choose not to complete work that will end with small effects that do not interest readers. Reviewers or editors might discount the value of a study that does not generate an exciting result. Such behavior by authors, reviewers, or editors could bias published impacts to be larger than the unbiased truth. A standard meta-analysis would normally rely on the effect sizes and their statistical significance reported in each study to conclude if studies with positive or larger results are likely to get published [5–7]. Unfortunately, these types of indicators are oftentimes unavailable in studies that utilized simulation models, raising a question about how to test for publication bias in the biofuel policy literature [8–10].

The primary contribution of this study is to introduce a new way to detect publication bias. Unlike these standard routes to use meta-analysis to detect bias, we test whether or not market simulation results are routed into one or the other of two narratives, food-versus-fuel or greenhouse gas (GHG) emissions, by the publication process. Observations from these two bodies of literature are not estimated coefficients that can be characterized in terms of at least two moments (e.g. mean and standard error) and our test is not designed to detect reasons for any bias. Our study, therefore, does not fit into a traditional meta-analysis or a systematic review whose methods have been well-established in health and medical sciences.

The food-versus-fuel debate revolved around the potential for biofuels to push agricultural commodity prices higher. Rising ethanol production corresponded to a rise in agricultural commodity prices, including the price of the most commonly used ethanol feedstock in the U.S., corn. We do not make claims about the causal factors of that price surge, but we recall that popular press accounts sometimes portrayed events as a stark trade-off between fuel and food security at that time. Legislation that created the U.S. biofuel mandates includes conditions that are intended to limit the food price impacts of biofuels. In academic work, this debate relates to an empirical scientific question as to the extent that rising demand for corn to make into ethanol increases the corn price [11]. If publication bias is present because of a systemic preference for big numbers or if anti-ethanol scientists seek to stress the risk of higher food prices, then results from models generating large price effects would be more likely to become food-versus-fuel publications than studies that generate smaller corn price impacts. What constitutes a big or large price effect might be partly subjective, but assessments might be based on the size of impacts in other studies, the perceived likelihood of being referenced, or an impression of what is relevant to policy making,

The GHG debate relating to ethanol rests in part on the potential for rising ethanol-related demand for corn to cause land use changes that release climate-changing GHGs. An argument is that greater ethanol use in the United States raises corn prices, so area shifts from other crops to corn and all crop prices will also rise, leading to expanding crop area in the United States, Brazil, and elsewhere. Such indirect land use change could cause large GHG emissions. U.S. biofuel mandates have prohibitions on crops grown on newly farmed land from being used to produce qualifying biofuels. Yet biofuel also must meet GHG reduction targets to qualify. The debate about the environmental outcomes of biofuels has direct policy relevance and many academic studies have attempted to estimate the impact of biofuels on GHG emissions. If publication bias is present because of a systematic preference for big numbers or if anti-ethanol scientists seek to support this narrative, then models generating large area effects would be over-represented in published scientific estimates of ethanol GHG emissions. Bias would favor studies that estimate large GHG emissions. Again, the definition of large and small effects might be based on other studies, perceived policy relevance, or other criteria. Perhaps, we argue below, a way to tell what sort of price effect is large or small would be to compare between the two bodies of literature. Because simulation models with large land area effects tend to have smaller price effects, we can use commonly reported price impacts to compare these two bodies of literature.

Another contribution is to introduce measures of competing narrative as an input to detect publication bias. A number of studies have used text and content analysis to detect thematic differences in, for example, how keyword usage evolved over time in climate science literature [12], whether abstracts written in a narrative style have more citations [13], or how sentiments varied in public responses to COVID-19 [14]. However, there is also a risk of misusing text mining for qualitative research as it relies largely on the quality of the text sources and the understanding of text mining process to ensure model assumptions correspond well to how topics are present in the corpus [15]. Here, we develop narrative indicators based on word counts. Specifically, we compare the frequency of words relating to food-versus-fuel literature to the frequency of GHG-related terms, or as a binary variable that reflects the presence or absence of a key numerical output relevant only to GHG studies.

The article is organized as follows. First, we set the context of publication bias and define the hypotheses we test. Second, we define our data and empirical tests. Third, we conduct analyses and explore sensitivities. Fourth, we discuss key results and state limitations. Finally, we draw certain conclusions from the study.

## 2. Materials and methods

The two ethanol debates allow us to test for certain types of publication bias (see Supplement for additional information). Our focus is the output of economic models with explicit market equilibrium conditions that are commonly used to estimate price and quantity impacts of policy or market shocks [16–19]. Certain economic simulation model characteristics that tend to cause large price changes will be associated with small area effects, and vice versa. Publication bias would tend to select models for these two debates based on these characteristics.

### 2.1 An illustration

We present a stylized representation here to illustrate how characteristics of economic models can cause systematic differences in price and land impacts that publication bias, if present, would tend to route into one of two bodies of literature.

**Fig 1:**
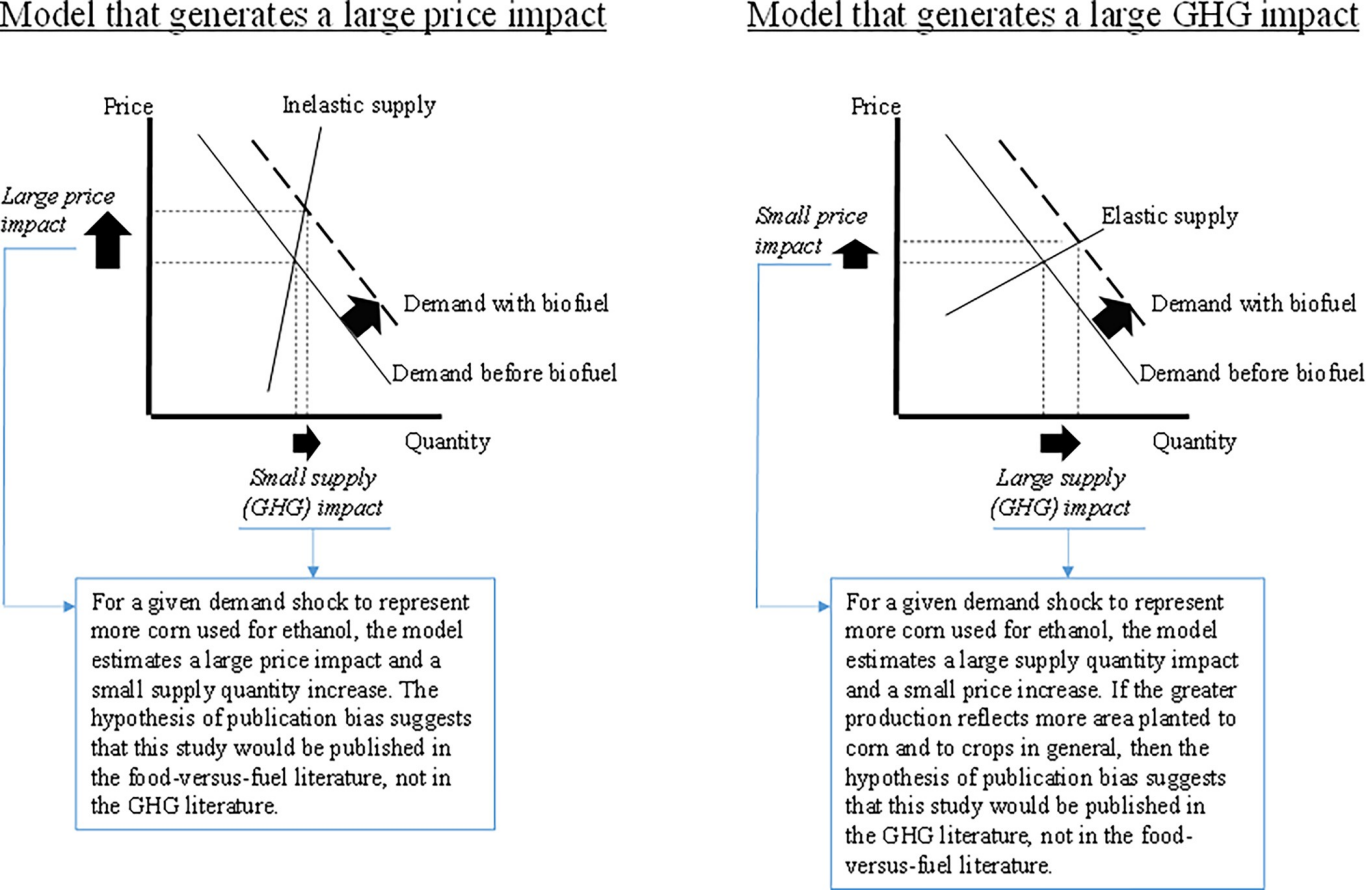
Publication bias implies that models simulating the impact of ethanol on corn markets would be sorted into different bodies of literature based on their fundamental characteristics.

Consider two economic models that are alike in every way except the elasticity of crop area (Fig 1). One model represents crop area and total crop supply as less responsive to price, or inelastic (left-hand graph). Crop area and crop supply are more responsive to price, elastic, in the second model (right-hand side). For a given demand shock, these two models will give different outcomes: the model with inelastic supply will estimate larger price impacts than the model with elastic supply; and the model with elastic supply will estimate larger supply and area impacts than the model with inelastic supply. Because land use change can be a key contributor to GHG emissions [20–23], the land use expansion of this latter model response will tend to be associated with greater GHG emissions, all else equal. Publication bias in favor of big effects would select the large price effect (and small land use effect) from the model with inelastic supply for dissemination as a food-versus-fuel study and select the large GHG effect (and small price effect) from the model with elastic supply for dissemination in the GHG literature. Big and small can be considered as relative indicators across the two narratives: if this form of publication bias is present, then a “big” price effect that is close to the average value among the food-versus-fuel study findings would tend to be larger than the corn price changes seen in the GHG literature A well-constructed test could detect if there are differences in the simulated price effects reported in the food-versus-fuel literature and GHG emission literature.

Continuing with a stylized representation illustrates how bias could be tested by comparing published price effects from economic models. Suppose that a crop model can be summarized based on the global demand, area, and yield response to crop prices. Assume that the own-price elasticities of demand (*ε*_*d*_), area (*ε*_*a*_), and yield (*ε*_*y*_) have the following characteristics:

$${\varepsilon_{d}\in [-\infty ,0],\;\varepsilon }_{d}∼N(-0.1,0.0025);$$ (1)

$${\varepsilon_{a}\in [0,\infty ),\;\varepsilon }_{a}∼N(0.2,0.01);\;and$$ (2)

$${\epsilon_{y}\in [0,\infty ),\;\varepsilon }_{y}∼N(0.1,0.0025).$$ (3)

**Fig 2:**
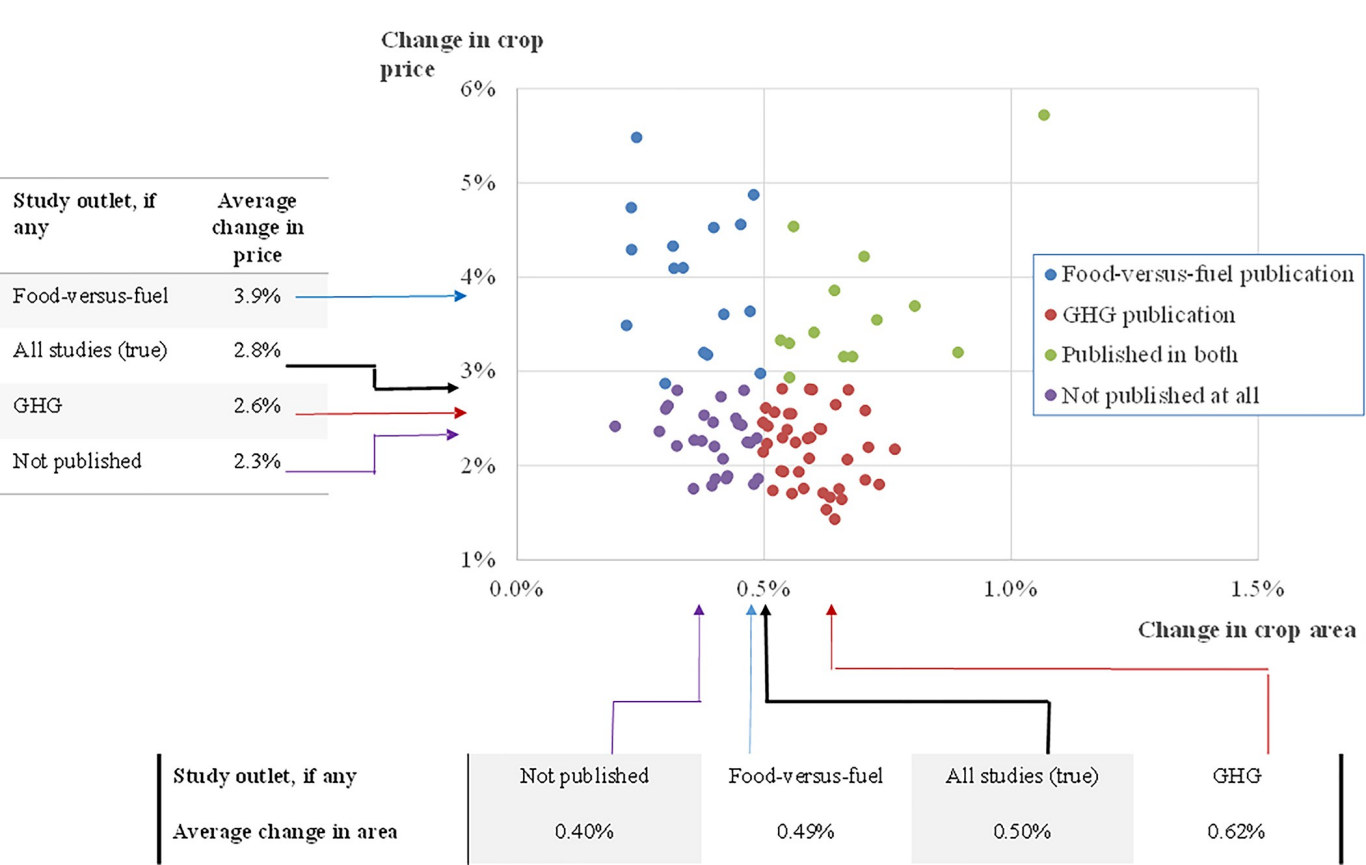
Hypothetical distributions of food-versus-fuel and GHG studies relative to the average of all studies and unpublished studies if bias disallows publication of below average effects.

A series of random draws on these variables reproduces a set of models that could be used to analyze a demand shock (Fig 2). A demand shock is assumed to be 1% in all instances, and the associated price impact is calculated based on these elasticities. The area effect is calculated based on the price change and the area elasticity. If publication bias is assumed to disallow the publication of any study that has below-average impact, then the sets of observations are selected for one of three outlets, GHG, food-versus-fuel, or both, or not published at all. These ranges for key parameters and this representation of publication bias lead to different results in the two bodies of literature (Fig 2). For example, the average price effect of all studies is 2.8%, but the average of food-versus-fuel studies is 3.9% and the average price effect in GHG studies is 2.6%. A bias towards publishing large results would mean the studies with “big” price changes would generate a 3.9% average impact in the food-versus-fuel literature, greater than the 2.6% average price impact apparent in the GHG literature. As for area effects, which at least some authors argue might have important GHG consequences, the GHG studies would average just over 0.6%, whereas all studies and food-versus-fuel studies average about 0.5%. In this hypothetical example, because it imposes the rule that below-average effects do not get published, unpublished studies have the lowest price and area impacts.

### 2.2. Publication bias hypotheses

**Fig 3:**
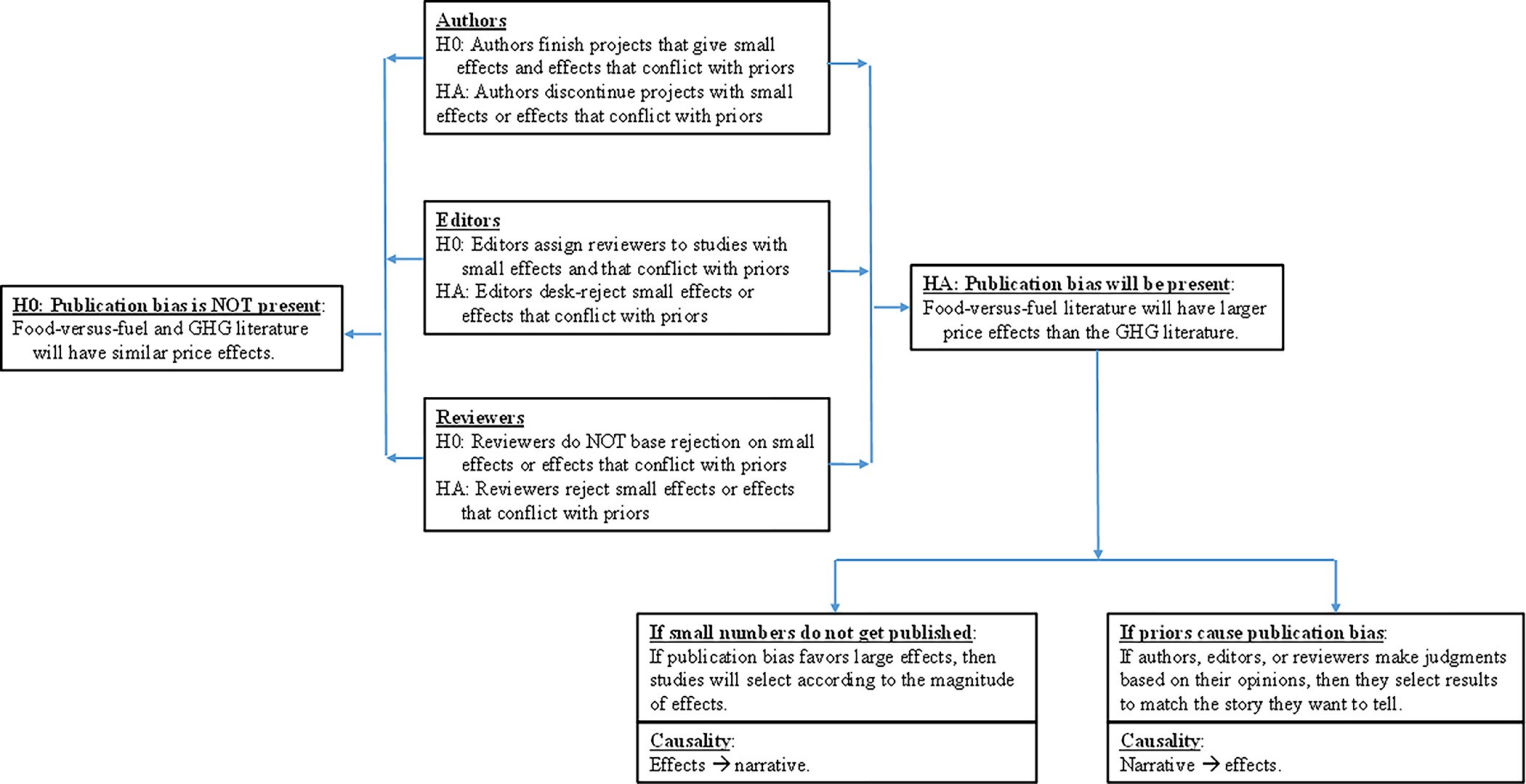
Developing null and alternative hypotheses of publication bias in the ethanol literature.

Our goal is to test for publication bias in simulation model results with potentially complicated causal relationships. We identify forms of publication bias and the implied causal relationships (Fig 3). Our null hypothesis is that there is no publication bias. The alternative hypothesis is that publication bias exists. Our empirical approach recognizes that publication bias can take different forms.

Publication bias might be caused by the maxim, “small numbers do not get published,” or else by prior expectations or personal views on the parts of the humans whose decisions define what gets published. These two causes lead to different tests. If publication bias favors big effects, then we would expect that scientific studies will be selected for publication only on a topic, or in a body of literature, where the results of the study appear to be large. Thus, a study with a large price effect and small area impact would tend to be published in the food-versus-fuel literature whereas a study with a large area effect and a small price effect would tend to be published in the GHG literature. Publication bias relating to size of impacts implies that the estimated effects determine the narrative.

The second hypothetical explanation for the alternative hypothesis is that human bias causes publication bias, in that authors, editors, and reviewers tend only to publish studies with effects that are aligned with their priors. If scientists tend to be anti-ethanol and their views affect their work, then this prior might be manifested as a preference for big price effects in the food-versus-fuel literature and big area effects in the GHG literature. If scientific results are affected by a pro-ethanol bias, then the results would tend to go in the opposite direction. In reality, the exact reason for the priors among authors, reviewers, and editors is not the point. Instead, the question is if their beliefs drive bias in scientific publications. Such forms of publication bias would mean that the narrative determines the published effects.

Our empirical approach follows from these definitions of null and alternative hypothesis. First, if publication bias is a consequence of the search for big effects, then the estimated change in corn price or land use will lead the study to be published in either food-versus-fuel or GHG literatures. In this case, the dependent variable is the narrative indicator. The explanatory variables will include measures of the size of the price impact. If the size of the price impact has a significant effect on the degree to which a study targets the food-versus-fuel literature, all else equal, then this finding would provide evidence of publication bias. This sort of result could imply that small numbers do not get published. The regression equation is

$$W_{j}=\alpha_{0}+\alpha_{1}P_{j}+\alpha_{3}R_{j}+\alpha_{4}P_{j}R_{j},$$ (4)

where the narrative indicator (*W*), for each study *j* is related to the indicator of corn price change, (*P*), and a dummy that takes a value of one if the study was published in a journal and is zero otherwise (*R*). There is only one narrative observation for each study, so in instances where studies have many estimates, we test this relationship using the average or the maximum estimated price impact. A statistically significant coefficient on price effect would be evidence of this type of publication bias.

Second, if publication bias is a consequence of the priors of humans who decide what gets published, then the narrative will be an independent variable that helps to explain the size of the estimated impacts. If a large narrative variable indicates that the study is published in the food-versus-fuel debate literature and if the coefficient on this variable is positive, then this finding would provide evidence of publication bias caused by human bias.

Regression equations can be specified that test for this form of publication bias. The regression equations to detect publication bias that could be regressed over all estimated price effects, *P*_*is*_, of each scenario, *i*, and of each study, *s*, are

$$P_{is}=\beta_{a0}+\beta_{a1}W_{s}+\beta_{a3}R_{s}+\beta_{a4}W_{s}R_{s,}\;\mathrm{or}$$ (5A)

$$P_{is}=\beta_{c0}+\beta_{c1}W_{s}+\beta_{c2}R_{s}+\beta_{c3}W_{s}R_{s}+\beta_{c4}M_{s}(W)+\beta_{c5}^{A}S_{is}^{A}(W)+\beta_{c5}^{B}S_{is}^{B}(W),$$ (5B)

where the price effect is a function of narrative (*W*), the dummy relating to publication in a journal (*R*), model type dummies (*M*), and scenario design characteristics (*S*), reflecting findings in previous research that the initial level of ethanol and the size of the change influence the price effect [24,25]. The first version of this Eq (5A) tests if word count affects price effect, without seeing if this effect is by directly routing results to each literature or by selecting models and scenarios to achieve a goal. This is a general test of the relationship that sacrifices explanatory power in the absence of controls yet focuses attention on the role of narrative. The second version of this Eq (5B) adds control variables and requires supplemental tests to determine if certain control variables are themselves functions of the narrative. We test these two equations. A statistically significant effect of narrative on price effect (in Eqs 5A or 5B) or on controls would be evidence of this type of publication bias (in Eq 5B).

## 3. Data

### 3.1. Data sources

The dataset begins with two reviews of the literature on the corn price impact of corn starch ethanol. We focus on the period when the two debates seemed most active, which runs from approximately the start to the run-up in commodity prices and introduction of the United States biofuel use mandates in 2005 until 2022 (see Supplement for additional information) [24] report that they identify 157 observations from 29 studies (p. 65) [25] review 173 studies published since 2010, from which 66 observations of the corn price change are drawn (p. 6, 12). Both reviews adopt the ratio of the corn price change to the ethanol volume change as a key indicator. For example, [24] report an average impact among studies of +2.9% corn price per billion gallons of corn starch ethanol (p. 66) and [25] report that the median estimated impact is +0.15 USD per bushel for each billion gallons of corn starch ethanol under certain conditions (p. 12).

Both reviews identify any number of potential explanatory factors that can influence the size of the corn price change per billion gallons of ethanol, either through a formal meta-analysis or by simple comparisons. The initial level of corn starch ethanol and size of change play a role, to give two examples. Model characteristics and dissemination outlet also appear to explain some differences among estimated impacts. For example, [25] report +0.15 USD per bushel of corn for a change in corn starch ethanol, but also show a greater effect if the sample includes short-run studies without United States area response and a lower effect if the sample includes studies that simultaneously shock other biofuel volumes rather than United States corn starch ethanol in isolation. [24] find statistically significant effects of model characteristics, dissemination outlet, and size and direction of the change in corn starch ethanol, as well as the presence of a policy instrument other than the biofuel mandate. The findings of these previous studies partly inform our empirical strategy.

We merged data from these two reviews and then extended them by including more studies. We searched for published and unpublished studies (reports, dissertations and theses, unpublished manuscripts) in the ethanol literature using a combination of keywords to identify relevant studies. This collection of studies represents the foundational set for our data, but we sought to include all relevant studies. We searched different academic databases including Google Scholar, EconLit and EBSCOhost. Studies were also identified by checking cross-references, authors’ websites and websites of research institutions in the field. The focus on certain types of new quantitative results prevented us from using from any commentaries, meta-analysis, or certain approaches as discussed further in the next section.

While we reviewed studies of price effects using other techniques, such as time series estimation, these techniques do not generate GHG impacts so there is no empirical test that allows us to compare estimates across two lines of literature. Thus, we only use results from structural or equilibrium economic models that can generate both price and GHG impacts of ethanol.

### 3.2. Restriction to corn starch ethanol data

First, studies that shock multiple biofuels or several instruments can give misleading estimates of the corn price impact of ethanol. For example, suppose an experiment increases all grain-based ethanol and biodiesel from vegetable oils in the United States and the European Union. In this case, the experiment design could cause a larger direct demand shock to soybean oil and soybean markets than corn markets, so the increase in the United States corn price could be driven more by competition with soybean for land than by the demand-side effects owing to ethanol. A ratio of the change in corn price to the change in corn starch ethanol would not measure the impact of ethanol on corn markets in this case. We focus on market simulation experiments where results are driven by corn ethanol to eliminate such errors.

The second risk of including studies that change more than corn starch ethanol alone is that publication bias could interact with these effects. If cross-effects from other biofuels, such as European biodiesel or Brazilian ethanol, would tend to cause corn price to increase by more or less, then publication bias would tend to cause studies that have certain scenario definitions to be sorted into one literature or the other. Whereas we test for other risks that study design is influenced by publication bias, we do not at this time see a way to test reliably for the possibility that such cross-effects bias published works.

### 3.3. Narrative data

Word counts are indicators of how final results are narrated and disseminated, so we use word counts or the presence of GHG results to assign studies to one literature or the other. We count the number of times authors use certain terms relating to the food-versus-fuel debate (“food”, “price”) and the number of times authors use other terms that relate to the GHG debate (“greenhouse gas”, “GHG”, “land”, “ILUC”). We also count the total number of words in the title, abstract, and main body. The counts exclude references. Two word-count indicators of narrative used in regression analysis are (1) the difference between food-related words and GHG-related words, divided by total words, and (2) the difference between the incidence of “food” and “GHG”, divided by total word count.

Word count indicators are appropriate if there are degrees of publication bias, with a relationship between larger estimated effects and more one-sided narratives. However, we also conduct empirical estimation with a binary measure that takes a value of one if the study generates GHG estimates and zero otherwise. Food-versus-fuel articles do not report GHG estimates, given the computation costs, space limitations of articles, and the irrelevance of these numbers to that narrative, so this binary variable can assign articles to the two bodies of literature.

### 3.4. Data summary

Out of 519 studies found in our review, we identify 38 articles and reports that provide a total of 221 estimates of the impact of corn starch ethanol on the corn price in the United States, including those that focus on aggregate coarse grains instead of corn alone. Many studies have more than one scenario or simulation, although we exclude any simulation result in which authors place no credence. For example, if authors present partial impacts or a stepwise process to build up to their best estimate of the total effect, then the intermediate results are not the authors’ estimated market impacts and these outcomes are omitted. More generally, we exploit simulation results to support our work. In some cases, authors present no single set of results that identifies the impacts of corn starch ethanol, but we can compare among scenarios in order to calculate the indicators that we need, even if the authors did not do so in the original publication.

Our dataset of estimated corn price effects tied strictly to corn starch ethanol quantity has the following attributes (Table 1). The simple average price change over all observations is $0.76 and the average is $0.64 if each study is given equal weight, regardless of the number of simulated price effects reported. The ratio of corn price change, in level and in percentage, per billion gallon increase in corn starch ethanol averages $0.23 and 5.19% over all estimates. If the estimates of each study are weighted so that each study counts as a single observation, to avoid putting too much emphasis on studies that report more outcomes than others, then the average corn price impact per billion gallon increase in corn starch ethanol is $0.24 or 4.68%. These numbers are at least somewhat higher than the averages reported by [24,25]. The initial level of corn starch ethanol varies widely, from zero to well over the 15 billion gallon potential limit of applicability to the U.S. Renewable Fuel Standard (RFS), and the range in ethanol quantity changes is even greater. Indeed, these wide ranges of initial values cause an even wider range in the percent change in corn starch ethanol, leading us to use the absolute change in corn starch ethanol as the denominator of the key ratio we use to measure the price-per-ethanol impact.

**Table 1:** Statistics of studies that generate directly useful price estimates.

						Price effect indicator		Narrative indicator	
Study	Public-ation year	No. of estimates	Initial level of corn starch ethanol (b.g.)	Average level of corn ethanol increase (b.g.)	Average corn price change ($/bu.)	Average ratio of corn price change ($/bu.) per billion gallon increase in corn ethanol	Average ratio of corn price change (%) per billion gallon increase in corn ethanol	Share of food-related vs. GHG-related word count in total word count (%)	Share of food vs. GHG word count in total word count (%)
Elobeid et al.	2007	1	19.1	24.6	1.38	0.06	1.67	2.37	0.02
Park & Fortenbery*	2007	1	5.0	5.0	0.51	0.10	3.20	2.04	0.07
Babcock	2008	6	17.1	4.4	0.33	0.08	2.12	1.88	0.17
de Gorter & Just (a)	2008	2	10.0	3.3	0.51	0.15	4.86	1.92	-0.04
Elobeid & Tokgoz	2008	2	7.1	0.6	0.05	0.08	3.23	2.01	0.08
McPhail & Babcock (a)*	2008	4	7.6	3.1	0.79	0.27	5.78	2.52	0.10
McPhail & Babcock (b)*	2008	4	9.8	0.5	0.33	0.68	12.85	1.83	0.22
Searchinger et. al**	2008	1	14.8	14.8	1.27	0.09	2.73	-4.23	-0.56
de Gorter & Just (b)	2008	2	9.0	3.0	0.49	0.16	4.90	1.88	0.13
Tyner & Taheripour	2008	76	10.9	7.1	0.88	0.14	5.09	2.12	0.02
Thompson et al.	2009	1	17.7	4.7	0.28	0.06	1.50	2.94	0.00
Babcock et al. *	2010	15	12.6	1.5	0.52	0.34	10.79	1.85	0.03
Fabiosa et al.	2010	1	10.3	1.0	0.09	0.09	2.79	0.41	0.15
Gohin & Treguer	2010	2	0.0	5.0	0.52	0.10	3.84	1.97	0.25
Babcock	2011	5	8.2	1.2	0.12	0.11	3.69	2.90	0.23
Cui et al.**	2011	5	0.1	13.1	1.59	0.12	4.99	0.89	0.00
Babcock & Fabiosa	2011	1	8.2	1.9	0.13	0.07	2.08	2.92	0.26
Babcock*	2012	1	13.3	1.3	1.91	1.47	15.10	2.47	0.04
Chen & Khanna	2012	1	12.3	1.6	0.68	0.41	6.17	0.89	0.60
Meyer & Thompson**	2012	1	15.6	4.0	0.62	0.15	3.27	0.58	-0.34
Thompson et al. *	2012	2	12.2	0.2	0.05	0.33	4.24	1.45	-0.04
Tyner et al. *	2012	3	13.8	6.1	2.00	0.33	4.27	2.06	0.40
Babcock & Zhou	2013	7	14.7	1.5	0.25	0.25	5.41	2.37	-0.02
Roberts & Schlenker	2013	1	0.0	11.0	1.07	0.10	2.73	2.23	0.31
Roberts & Tran	2013	30	13.6	4.1	0.96	0.28	3.49	3.00	0.12
Bento & Klotz**	2014	4	9.5	4.7	1.20	0.26	9.07	-0.77	-0.55
Gohin**	2014	3	1.8	13.3	0.29	0.02	1.12	0.62	-0.14
Herreros et al.	2014	1	13.3	0.8	0.15	0.19	2.72	1.24	0.02
Wu & Langpap	2014	3	0.0	28.5	2.52	0.09	2.10	2.46	0.77
Dhoubhadel et al.	2015	1	13.8	1.9	0.38	0.20	2.91	0.88	0.02
Bento et al.**	2015	4	11.8	2.3	0.54	0.24	7.27	-0.98	-0.04
Jones & McCarl*	2016	9	11.9	1.0	0.48	0.48	5.15	1.18	0.03
Jones et al.*	2017	10	12.2	1.6	0.83	0.44	5.01	1.16	-0.03
Zhou & Babcock	2017	6	14.9	1.1	0.25	0.24	5.61	2.46	0.05
Chen & Khanna	2018	1	6.5	6.7	1.31	0.20	2.84	0.75	0.23
Perez-Pena & Palaez-Herreros	2019	1	7.6	0.1	0.02	0.29	8.06	0.81	0.06
Schmitz et al.	2020	1	0.0	0.0	-1.12	.	.	2.19	0.03
Taheripour et al.	2022	2	13.5	1.2	0.02	0.02	0.50	1.87	0.27
Simple average			11.1	5.1	0.76	0.23	5.19	1.94	0.05
Study-weighted average		5.8	10.0	4.9	0.64	0.24	4.68	1.50	0.08
Number of observations		221	221	221	221	221	221	38	38

Notes: B.g. denotes billion gallons. Bu. denotes bushel. An asterisk (*) indicates a short-run study that we define as focusing on immediate impacts of an ethanol quantity change before United States area can respond to any price effect and a double asterisk (**) indicates a study that estimated GHG effects of ethanol. Further information about these studies, including the criteria for including a study, is available in the nearby text. A full list of references is provided in the Supplement, section S4.

**Fig 4:**
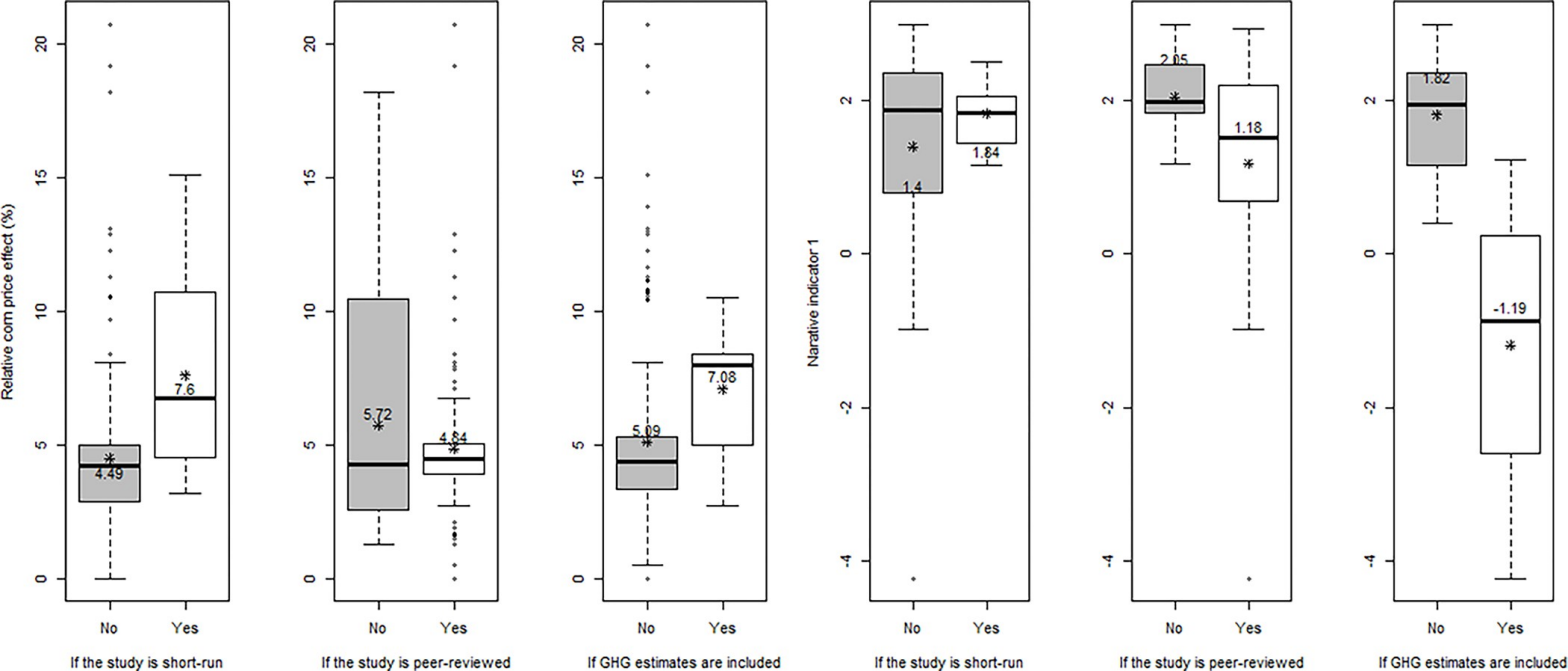
Box-and-whisker plots of corn price effect and narrative indicator by study characteristics. Notes: (1) Relative corn price effect is the average ratio of corn price change (%) per billion gallon increase in corn ethanol; and Narrative indicator 1 is the share of food-related words versus GHG-related words in the total word count. (2) The left and right edges of the box represent the 25^th^ and 75^th^ percentiles (interquartile range); the line within the box represents the median; the asterisk represents the mean value; the left and right whiskers are defined using a standard coefficient of 1.5 times the interquartile range; and outliers are not shown. (3) Box in white indicates if the answer is Yes; box in gray indicates if the answer is No.

Comparing certain subsets to the total suggest some differences, many of which are anticipated (Fig 4). The difference between price effects is more striking if comparing studies focused on short-run impacts, defined as studies without U.S. area response, and studies that allow more time for adjustment. Short-run studies are associated more with the food-versus-fuel debate than with GHG impacts when judged by our narrative indicators tied to word count. The difference in narrative among peer-reviewed and other publications is smaller. Additional details are available in a supplement. For example, as regards model type, general equilibrium models account for 7% of the estimates. Partial equilibrium models are the basis for the remaining 93% of the estimates, and these studies reflect a wide variety of models by many different authors. The average publication dates of all subsets are reassuringly similar, suggesting that differences among groups of studies might not reflect other factors, like growing scientific awareness and increased accuracy over time.

**Fig 5:**
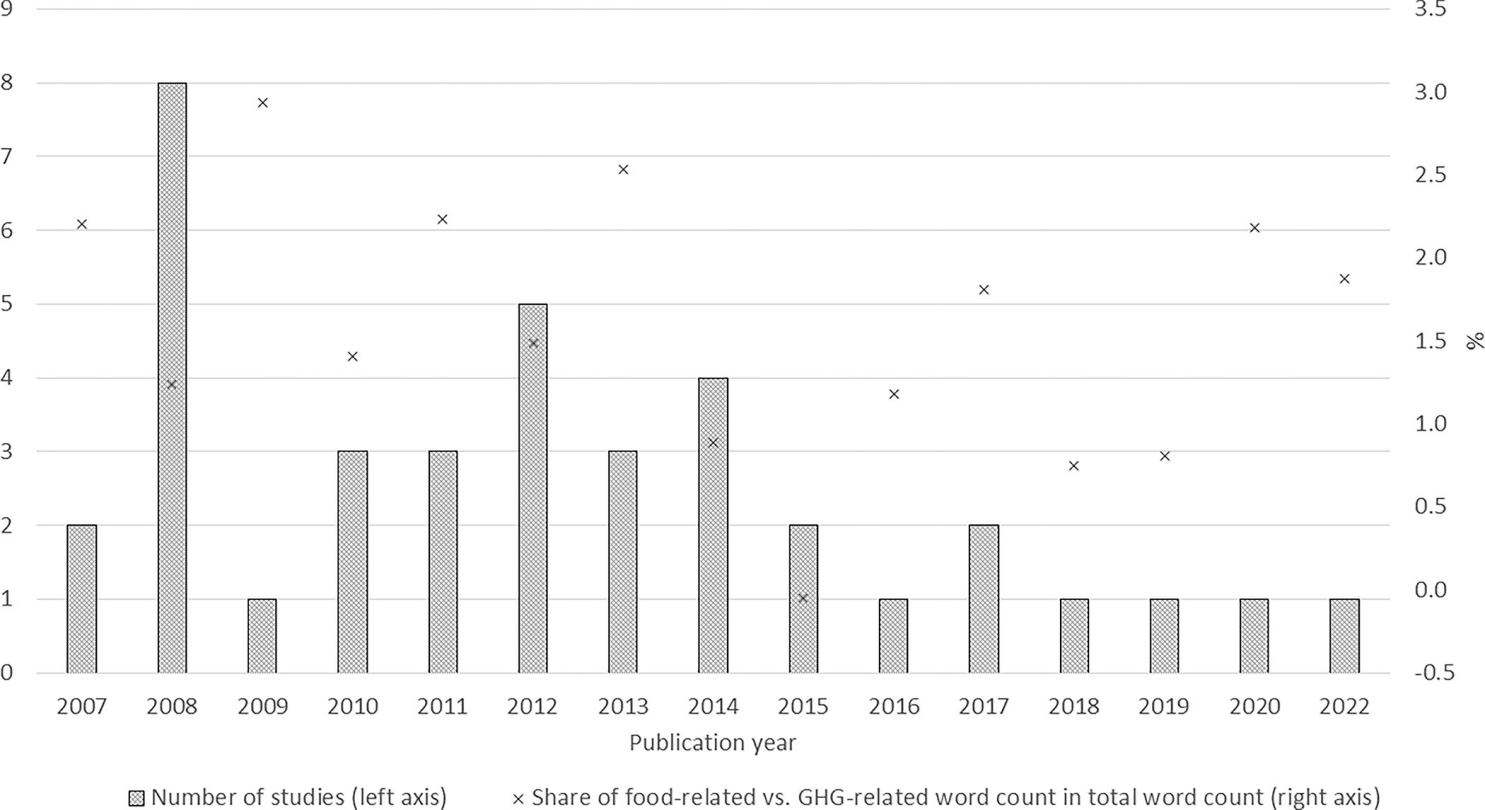
Number of studies and narrative indicator by year.

The narrative indicator that compared food- and GHG-related word counts relative to total words ranges from -4% to +3%, with an average of 1.9%. A comparison of this indicator and number of studies by year suggests an unsteady tendency for fewer food-versus-fuel articles over the fifteen-year period with publications becoming generally less frequent after 2014 (Fig 5). The correlation of the two word-count variables is 0.6.

### 3.5. Estimation

Observations of the dependent variable of Eq 5 are not independent as we find about half a dozen estimates drawn from each study, on average. In such cases, fixed-effects and random-effects models are the main approaches to address the issue of inter-dependence in multilevel data [26]. [24] used both approaches and preferred the fixed-effects model as it provided more consistent results. Nevertheless, we found that a fixed-effects model has a limitation in estimating the effects of group-level variables (i.e., variables that do not vary within a group such as a dummy indicating if a study uses a general equilibrium model). We opt for a mixed-effects regression for Eq 5 to account for both fixed and random effects. Whereas previous researchers might opt to group observations by model type (e.g., [24]), we group observations by study to take into account the fact that multiple observations are drawn from many studies and to try to reduce the risk that study-specific effects other than publication bias could be associated with our key word count variable. We do not assume that the impact of word count on price effects is the same across studies. In the mixed effect regressions, we apply models with random intercepts and random slopes on the word count variable, meaning that the word count variable takes a different coefficient estimate for each study. For the narrative binary variable, we apply a random intercept regression instead.

The estimation method for Eq 4 is less complicated because we have one observation of the dependent variable, word count, from each study. We run additional regressions to test for the sensitivity of results to alternative proxies for narrative and price effect. For example, although the corn price impact indicators are informed by [24,25] to standardize study results, our tests for bias against small effects also use the corn price effect directly, without division by the volume of ethanol change.

We recognize that this is a new method to test for publication bias and explore several possible limitations with additional regression analyses. Sensitivity analysis addresses some possible concerns, including those relating to the exact choice of narrative variable, clustering, and endogeneity, to some extent.

## 4. Results and discussion

### 4.1. Results

The regressions of the narrative as a function of price change indicator and other variables (Eq 4) find little or no evidence to support the view that publication bias is caused by such factors as the suppression of small numbers or a preference for small numbers (Table 2, Table 3, and supplement). The coefficients associated with price change, either alone or in combination with the refereed journal dummy are not statistically significant, with a 5% critical value, so there is no evidence of publication bias whereby the results drive the text. This result is not consistent with “small results do not get published”, for example, nor with a view that small results are more likely to be published.

**Table 2:** Word count as a function of price change indicator (average values by study).

	Narrative indicator 1		
	(1)	(2)	(3)
Model 1			
Average price change	-0.21	-0.198	0.185
	(0.332)	(0.314)	(0.574)
Journal publication (Yes = 1, No = 0)		-0.980*	-0.634
		(0.430)	(0.612)
Average price change x Journal publication			-0.549
			(0.687)
Constant	1.637***	2.248***	2.010***
	(0.304)	(0.394)	(0.496)
R^2^	0.011	0.139	0.155
Adjusted R^2^	-0.016	0.09	0.08
Model 2			
Semi-elasticity price change	-0.011	-0.071	-0.01
	(0.073)	(0.071)	(0.088)
Journal publication (Yes = 1, No = 0)		-1.178*	-0.366
		(0.456)	(0.823)
Semi-elasticity price change x Journal publication			-0.176
			(0.149)
Constant	1.538***	2.551***	2.182**
	(0.408)	(0.545)	(0.625)
R^2^	0.001	0.164	0.198
Adjusted R^2^	-0.028	0.115	0.126
Observations	37	37	37

Notes:

* p < .05

** p < .01

*** p < .001. Narrative indicator 1 = Share of food-related vs. GHG-related words in total word count. Standard errors are in parentheses.

**Table 3:** Word count as a function of price change indicator (maximum values by study).

	Narrative indicator 1		
	(1)	(2)	(3)
Model 1			
Maximum corn price change	-0.052	-0.021	0.27
	(0.210)	(0.199)	(0.461)
Journal publication (Yes = 1, No = 0)		-0.981*	-0.661
		(0.433)	(0.631)
Maximum corn price change x Journal publication			-0.36
			(0.512)
Constant	1.553***	2.143***	1.892**
	(0.298)	(0.384)	(0.527)
R^2^	0.002	0.129	0.142
Adjusted R^2^	-0.026	0.079	0.066
Model 2			
Maximum semi-elasticity corn price change	0.041	0.006	0.03
	(0.047)	(0.047)	(0.071)
Journal publication (Yes = 1, No = 0)		-1.010*	-0.706
		(0.466)	(0.809)
Maximum semi-elasticity corn price change e x Journal publication			-0.045
			(0.096)
Constant	1.228**	2.076***	1.873*
	(0.368)	(0.525)	(0.690)
R^2^	0.022	0.14	0.146
Adjusted R^2^	-0.006	0.09	0.068
Observations	37	37	37

Notes:

* p < .05

** p < .01

*** p < .001. Narrative indicator 1 = Share of food-related vs. GHG-related words in total word count. Standard errors are in parentheses.

Regressions investigate the potential that publication bias of this second type, with bias causing results, is manifested in model selection or scenario design (Eq 5B). Empirical tests suggest that the narrative does not affect the starting amount of ethanol or the size of the change, not when assessed by the statistical significance of the coefficient on narrative in regressions with these controls as dependent variables (Table 4). Graphical inspection does not suggest a strong relationship between the word counts of studies and the effects of authors’ chosen models on price impact of ethanol (supplement). Consistent with the overall relationship defined earlier (Eq 5A), we test if the narrative variable takes on statistical significance in the absence of any controls (left-most columns of Tables 5 and 6). While this test sacrifices precision, the statistically insignificant coefficient on narrative suggests that this variable does not have a combination of direct and indirect effects on price through the controls in other regressions.

**Table 4:** Summary of supplemental empirical tests (Eq 5B) if publication bias affects control variables.

	Corn ethanol change (b.g.)		Initial level of corn ethanol (b.g.)	
	(1)	(2)	(3)	(4)
**Random Intercept and Random Slope for the Word Count Variable**				
Narrative	0.872	1.273	-0.331	-0.808
	(0.623)	(2.951)	(0.226)	(2.303)
Narrative*Journal		-0.419		0.276
		(3.019)		(2.394)
**Random Intercept Only**				
Narrative	0.099	0.951	-0.375	-0.664
	(0.793)	(2.678)	(0.714)	(2.129)
Narrative*Journal		-0.934		0.325
		(2.803)		(2.260)
**Random Intercept Only and with a Short-Run Dummy**				
Narrative	0.064	0.628	-0.367	-0.66
	(0.796)	(2.896)	(0.724)	(2.412)
Narrative*Journal		-0.611		0.321
		(3.012)		(2.526)
**Random Intercept Only and with Model Type Dummies**				
Narrative	0.91	-0.939	-0.68	-0.803
	(0.785)	(2.731)	(0.766)	(2.383)
Narrative*Journal		2.079		0.141
		(2.940)		(2.577)

**Table 5:** Absolute corn price change ($/bushel) as a function of word count.

	Corn price change ($/bu.)			
	(1)	(2)	(3)	(4)
(Intercept)	0.752***	0.37	-0.022	-1.016*
	(0.118)	(0.198)	(0.234)	(0.402)
Narrative indicator 1	-0.07	-0.069	-0.025	0.367*
	(0.064)	(0.070)	(0.071)	(0.145)
Journal publication (Yes = 1, No = 0)		-0.356*	-0.142	0.915*
		(0.152)	(0.161)	(0.412)
Initial level of corn ethanol (billion gallon)		0.017**	0.017**	0.018**
		(0.006)	(0.006)	(0.006)
Corn ethanol change (billion gallon)		0.091***	0.116***	0.119***
		(0.008)	(0.015)	(0.015)
Decrease scenario (Yes = 1, No = 0)		0.011	0.065	0.079
		(0.138)	(0.127)	(0.111)
Corn ethanol change squared			-0.001	-0.001
			(0.001)	(0.001)
Short run model (Yes = 1, No = 0)			0.399*	0.582***
			(0.162)	(0.158)
Narrative indicator 1 x Journal publication				-0.449**
				(0.159)
Number of groups	38	38	38	38
Observations	221	221	221	221
Akaike information criterion (AIC)	387	303	299	295

Notes:

* p < .05

** p < .01

*** p < .001. Narrative indicator 1 = Share of food-related vs. GHG-related words in total word count. Standard errors are in parentheses.

**Table 6:** Corn price change (%) per billion gallon increase as a function of word count.

	Corn price change (%) per billion gallon increase in ethanol			
	(1)	(2)	(3)	(4)
(Intercept)	5.077***	10.095***	6.949***	5.089*
	(0.737)	(1.098)	(1.297)	(2.059)
Narrative indicator 1	-0.14	-0.362	0.445	1.44
	(0.373)	(0.346)	(0.515)	(0.996)
Journal publication (Yes = 1, No = 0)		-2.418*	-0.698	1.492
		(0.959)	(0.814)	(2.046)
Initial level of corn ethanol (billion gallons)		-0.144***	-0.141***	-0.140***
		(0.033)	(0.033)	(0.033)
Corn ethanol change (billion gallons)		-0.173***	-0.174***	-0.170***
		(0.048)	(0.048)	(0.048)
Decrease scenario (Yes = 1, No = 0)		-1.920*	-2.713***	-2.552***
		(0.838)	(0.712)	(0.722)
Short run model (Yes = 1, No = 0)			2.914***	3.018***
			(0.837)	(0.838)
Narrative indicator 1 x Journal Publication				-1.356
				(1.159)
Number of groups	37	37	37	37
Observations	220	220	220	220
Akaike information criterion (AIC)	1097	1070	1066	1066

Notes:

* p < .05

** p < .01

*** p < .001. Narrative indicator 1 = Share of food-related vs. GHG-related words in total word count. Standard errors are in parentheses.

The journal variable does not have statistically significant effects in most instances, including when crossed with the price or narrative variable. The lack of statistical significance of narrative and price coefficients with and without journal cross term indicates no evidence of bias overall and no evidence of bias created by the journal publication process. In contrast, given that coefficients on price or narrative are statistically insignificant, a statistically significant coefficient on the cross of that term and the journal variable would be consistent with the view that journals cause publication bias–but we do not find this result. We do not find evidence that supports a claim that journal processes create bias and, perhaps because we find no evidence of publication bias elsewhere, results at this time do not support a claim that journal processes mitigate publication bias.

### 4.2. Sensitivity

We attempt to address at least some possible concerns with additional regressions (Table 7, see Supplement for detail). Our results are not sensitive to alternative indicators of narrative or price effect, for example, there is no statistical evidence of a relationship between narrative and price using other measures of each variable. Clustering by model is an alternative to clustering by study. In our analysis, we emphasize study clusters because our narrative variables are defined on a per-study basis and are the focal point of our research question. Regressions that cluster results by model do not generate statistically significant evidence of publication bias. Some observations are from studies that consider short-run price effects, often associated in some way with the 2012/2013 drought. Regressions that omit these studies do not produce statistically significant evidence of publication bias.

**Table 7:** Sensitivity analysis of key coefficients and errors from regressions with all control variables.

Panel A: Regressions of narrative on corn price effect		
	**Coefficient**	**Standard error**
**Alternative narrative variables**		
Indicator 2	-0.07	0.09
GHG binary variable	-7.49	9.20
**Alternative price variable**		
Average semi-elasticity	-0.00	0.02
Maximum absolute effect	0.05	0.09
Maximum semi-elasticity	-0.00	0.01
**Alternative price and narrative variables**		
Binary and average semi-elasticity	-1.64	1.54
**Alternative specifications**		
Model fixed effects	-0.02	0.06
Clustering by model	-0.54	0.52
Omit short-run studies	-0.53	0.65
Panel B: Regressions of corn price effect on narrative		
	**Coefficient**	**Standard error**
**Alternative narrative variables**		
Indicator 2	1.38*	0.67
**Alternative price and narrative variables**		
Indicator 2 and average semi-elasticity	-1.95	4.3
Binary and average semi-elasticity	-0.12	0.51
**Alternative specifications**		
Using study average price effect	-0.97	1.99
Panel C: Tests for bias in model selection or scenario design		
	**Coefficient**	**Standard error**
**Narrative on ethanol volume change**		
Random intercept	0.88	0.62
Random intercept and slope	1.27	2.95
Random intercept, short-run dummy	0.63	2.89
Random intercept, model dummy	-0.94	2.73
Using only study average	0.91	2.91
**Narrative on initial ethanol volume**		
Random intercept	-0.55	0.63
Random intercept and slope	-0.80	2.30
Random intercept, short-run dummy	-0.66	2.41
Random intercept, model dummy	-0.80	2.3
Using only study average	-1.08	2.49

There is a risk of endogeneity, or simultaneity, in food price and narrative variables (Eqs 4 and 5). This risk is reduced by the fact that the two tests focus on different types of bias: regressions of narrative on price change test if small results do not get published (Eq 4), and regressions of price effects on narrative test if human bias causes results (Eq 5). One of the equations will have an endogenous variable on the right side if either type of bias is present, and both equations will have this problem if both alternative hypotheses are true.

### 4.3. Discussion

We focus here on key limitations. This is a new method to test for publication bias and we advise the reader to apply a degree of caution when drawing conclusions. Rather than statistically estimated coefficients, our data are drawn from economic simulations. We do not test for bias in a key parameter value (such as regression coefficients measuring the effect of a policy or health care intervention), explore the distribution of parameter estimates, or try to explain publication bias. We develop a test for the presence of publication bias and focus this article on applying it, but we certainly encourage other scientists to adopt this method and develop their own experiments.

Controls that might commonly appear in other approaches to publication bias are not useful here. A key challenge is to avoid any control that interferes with our ability to detect publication bias. For example, trying to include economic model parameters as controls is not helpful in this context. Economic model structure can vary widely and defy simple comparison. Trying to develop some measures of model responses introduces the risk of partial indicators that are misleading, subjective judgments by us that could introduce our own biases, and mistakes given the potentially complicated model simulations to develop these response measures. More importantly, these controls defy the purpose of our research. If publication bias is present, then these model characteristics could be a method of implementing this bias, rather than exogenous. For example, including some indicator of crop supply response as a control variable in the price regression risks taking as an exogenous factor a key aspect of models that is part of the problem. Supply response has a role in the price outcome, so the coefficient could be important, but this model characteristic is why a model’s results could be biased towards one body of literature or another and yet this effect would be buried in the coefficient on supply response. So while model characteristics might improve fit or some other indicator of statistical performance, they could diminish the ability to detect bias.

Control variables about study design can also interfere with our hypothesis testing. For example, if bias drives published price effects, then the bias could take the form of scenario selection, such as larger ethanol shock or certain initial values. We focus on corn starch impacts on corn price to avoid the risk that broader scenarios include other shocks than just corn starch ethanol that also drive the corn price outcome. This restriction could be revisited, perhaps even testing if scenario scope can also be affected by bias.

We omit information about studies or authors that might speak to their subjective views. The goal here is not to test for motivations of bias. We do not try to firmly and objectively classify all authors as pro- or anti-ethanol. We do not include any variables relating to the funding for the study or author disciplines. Critically, if publication bias is present then causal factors would be correlated with our variables of interest, with the possible outcome that our yes-or-no test for publication bias is weakened by an unrelated question to do with some possible causes. We do not say that these considerations are unimportant, but they are not our considerations here.

An extension that is consistent with our framework and could provide important information would be to use data based on initially submitted manuscripts (including those that are rejected) and versions before final editing. These data and appropriate empirical tests could test for evidence of publication bias at each specific stage. In principle, however, the final published result is what matters most, so our starting point is justifiable even though evidence of publication bias at a stage of the process by authors, reviewers, or editors would also have value.

## 5. Conclusions

Publication bias is critically important to science and to policy. If present, then scientific inquiry does not generate accurate understanding, so public policy can be misled into higher cost and less effective options, or even prove counter-productive relative to societal goals. We exploit the two lines of literature relating to United States corn starch ethanol, one relating to price and food effects and the other focusing on GHG impacts, to test for the presence of publication bias. To some extent, differences in area response among simulation models will tend to cause inverse relationships in the size of price and quantity impacts from a given demand shock associated with greater ethanol demand for corn. Consequently, models might tend to generate larger price impacts or large area impacts. If present, publication bias in favor of large results, for example, would tend to cause certain types of results to appear in one or the other of these bodies of literature. Publication bias to support certain anti- or pro-ethanol narratives would select results from simulation models that tend to generate certain price and area impacts into the food-versus-fuel or GHG literature.

We examine the relationship between economic market model price effects and the orientation of studies towards these two literatures to test for publication bias. Ideally, these tests could detect if these reasons cause food-versus-fuel literature price effects to be large and GHG literature price effects to be small due to larger land response with consequent emission effects. Studies are assigned to the food-versus-fuel literature or the GHG literature (a “narrative”) using either the incidence of words associated with either line of literature or a binary variable that represents the presence or absence of GHG effect estimates in the study. Tests attempt to detect either relationship, subject to the limitations, from results to the narrative or from the narrative to the results. The price effect is not a statistically significant factor influencing the narrative, and the narrative is not a statistically significant factor influencing estimated price impacts and, moreover, narrative does not appear to drive other factors that do affect price impacts, namely model selection and scenario design.

The study contributes to the publication bias literature by raising the possibility that findings can be compared among bodies of literature where difference in results can be tied to the underlying theory. Whereas many other studies use statistics and indicators of statistical significance, we compare economic simulation model results published in two lines of literature. If the result of no evidence is confirmed, then this finding alone would be an important contribution to the ethanol debate and about human science more generally. The method itself is also of consequence, even setting aside the results of this one case. For example, publication bias is evident in at least some statistical findings around the yes-or-no judgment of statistical significance [27]. Similarly, a negative correlation between effect size and sample size as well as a biased distribution of p-values are taken as indication of widespread publication bias in psychology studies that rely on statistical methods [28]. Our work uses simulation model results that do not generate p-values or binary judgments about statistical significance. Comparing evidence of publication bias between statistical and simulation methods could give information that affects how we do science.

## Supporting information

S1 File (DOCX)
