# Supplementary material for: Publication bias in simulation model studies: The case of ethanol literature
Source: PLoS One. 2023 May 4;18(5):e0284715. doi: 10.1371/journal.pone.0284715 (PMC10159346; doi:10.1371/journal.pone.0284715)
Supplement: S1 File — (DOCX) [file pone.0284715.s001.docx]

Supplement for Testing for Publication Bias in the Ethanol Literature

The following material presents (a) additional detail about meta-analysis data, (b) information to support the view that there are distinct bodies of literature, and (c) additional regression results to test sensitivity of results.

**S1. Additional details about the data**

The following information relates to the data generated from the literature.

These data define studies by various characteristics that are used in the regression, including the outlet and the model used.

Word counts are used to generate a proxy for the degree to which a study is oriented towards the food-versus-fuel debate or the GHG debate. These are narrative indicators. Of the two word count indicators, the preferred variable is a broader measure that takes into account more words.

An additional narrative indicator is a binary variable based on whether or not a study reports GHG emission estimates.

Table 1. Study outlets.

|  | **No. of studies** | **No. of estimates** | **Average estimates per study** |
| --- | --- | --- | --- |
| Published in a journal | 24 | 136 | 5.7 |
| Not published in a journal | 14 | 85 | 6.1 |

Table 2. Models used.

| **Model** | **No. of estimates** | **Share of total** |
| --- | --- | --- |
|  |  |  |
| GE, GTAP | 5 | 2% |
| GE, other | 11 | 5% |
| PE, FAPRI-ISU | 9 | 4% |
| PE, FAPRI-MU | 2 | 1% |
| PE, short run | 49 | 22% |
| PE, other | 145 | 66% |

Table 3. Summary statistics

|  |  | **All studies** | **Timeframe** | | **GHG estimates** | | **Peer-reviewed** | |
| --- | --- | --- | --- | --- | --- | --- | --- | --- |
|  |  |  | Short-run | Long-run | Included | Not included | Yes | No |
| Totals of these studies | |  |  |  |  |  |  |  |
|  | Number of studies | 38 | 9 | 29 | 4 | 34 | 24 | 14 |
|  | Number of observations | 221 | 49 | 172 | 10 | 211 | 135 | 86 |
| Average values of these studies | |  |  |  |  |  |  |  |
|  | Year of publication | 2011 | 2012 | 2011 | 2014 | 2011 | 2011 | 2012 |
|  | Initial level of corn starch ethanol (b.g) | 11.10 | 11.66 | 10.95 | 11.31 | 11.09 | 10.00 | 12.84 |
|  | Average level of corn ethanol increase (b.g) | 5.07 | 1.79 | 6.00 | 4.36 | 5.10 | 6.53 | 2.78 |
| Indicators of corn price effect per billion gallon ethanol increase | | |  |  |  |  |  |  |
|  | Absolute corn price effect ($/bu.) | 0.23 | 0.43 | 0.17 | 0.23 | 0.23 | 0.17 | 0.32 |
|  | Relative corn price effect (%) | 5.19 | 7.60 | 4.49 | 7.08 | 5.09 | 4.84 | 5.72 |
| Indicators of narrative relative to total word count (%) | | |  |  |  |  |  |  |
|  | Food-related less GHG-related | 1.94 | 1.65 | 2.03 | -1.00 | 2.08 | 1.74 | 2.27 |
|  | Food versus GHG | 0.05 | 0.06 | 0.05 | -0.29 | 0.07 | 0.03 | 0.09 |

In the figure below and the regressions, the narrative of each study is related to the price changes.

Figure 1. Narrative (word count) and corn price change for 221 estimates drawn from 38 studies.


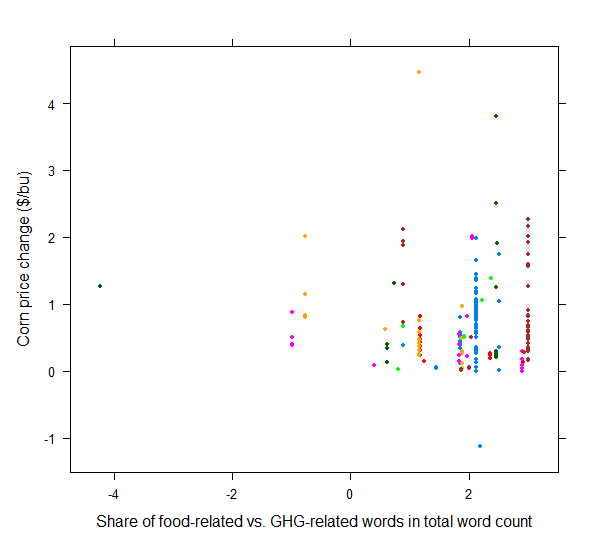


The word count and binary variables are critically important. We present these data. In reading this table, please recall definitions of words given in the main text and be aware that:

Word count 1 equals the difference between food-related and GHG-related word counts divided by total word count in percent; and

Word count 2 equals the difference in food and GHG word counts divided by total word count, in percent.

We also take the opportunity to note which studies are classified as short-run, meaning here that the focus is on the immediate price effects before United States area can respond

Notes follow the table.

Table 4: Word counts

| Study ID | Number of words appear in the title, abstract and main text | | | | | | Narrative indicators based on word counts | |
| --- | --- | --- | --- | --- | --- | --- | --- | --- |
|  | Total | "Food" | "Price" | "GHG" | "Land" | "ILUC" | Variable 1 | Variable 2 |
| 21 | 15642 | 4 | 285 | 0 | 0 | 0 | 1.85 | 0.03 |
| 36 | 4350 | 11 | 118 | 1 | 2 | 0 | 2.90 | 0.23 |
| 41 | 2224 | 1 | 54 | 0 | 0 | 0 | 2.47 | 0.04 |
| 44 | 4819 | 1 | 115 | 2 | 0 | 0 | 2.37 | -0.02 |
| 53 | 6502 | 8 | 30 | 44 | 44 | 0 | -0.77 | -0.55 |
| 64 | 4515 | 27 | 53 | 0 | 40 | 0 | 0.89 | 0.60 |
| 72 | 15222 | 12 | 149 | 12 | 9 | 5 | 0.89 | 0.00 |
| 78 | 10993 | 2 | 95 | 0 | 0 | 0 | 0.88 | 0.02 |
| 79 | 9572 | 15 | 154 | 1 | 129 | 0 | 0.41 | 0.15 |
| 90 | 6427 | 2 | 52 | 11 | 3 | 0 | 0.62 | -0.14 |
| 94 | 8082 | 8 | 101 | 6 | 3 | 0 | 1.24 | 0.02 |
| 110 | 7916 | 10 | 86 | 37 | 13 | 0 | 0.58 | -0.34 |
| 140 | 15660 | 48 | 333 | 0 | 32 | 0 | 2.23 | 0.31 |
| 141 | 9358 | 11 | 278 | 0 | 8 | 0 | 3.00 | 0.12 |
| 173 | 8110 | 2 | 125 | 5 | 4 | 0 | 1.45 | -0.04 |
| 177 | 5495 | 22 | 91 | 0 | 0 | 0 | 2.06 | 0.40 |
| 187 | 12140 | 94 | 212 | 1 | 5 | 1 | 2.46 | 0.77 |
| 202 | 4213 | 12 | 113 | 1 | 1 | 0 | 2.92 | 0.26 |
| 203 | 4618 | 9 | 79 | 1 | 0 | 0 | 1.88 | 0.17 |
| 210 | 11222 | 0 | 222 | 5 | 1 | 0 | 1.92 | -0.04 |
| 213 | 9172 | 7 | 180 | 0 | 3 | 0 | 2.01 | 0.08 |
| 219 | 6912 | 7 | 168 | 0 | 1 | 0 | 2.52 | 0.10 |
| 223 | 10193 | 25 | 165 | 3 | 0 | 0 | 1.83 | 0.22 |
| 227 | 2693 | 6 | 8 | 21 | 107 | 0 | -4.23 | -0.56 |
| 228 | 3811 | 0 | 112 | 0 | 0 | 0 | 2.94 | 0.00 |
| 231 | 7946 | 10 | 139 | 0 | 0 | 0 | 1.88 | 0.13 |
| 233 | 5103 | 1 | 124 | 0 | 4 | 0 | 2.37 | 0.02 |
| 240 | 5348 | 4 | 105 | 0 | 0 | 0 | 2.04 | 0.07 |
| 241 | 4330 | 1 | 92 | 0 | 1 | 0 | 2.12 | 0.02 |
| 317 | 6745 | 17 | 130 | 0 | 14 | 0 | 1.97 | 0.25 |
| 323 | 8185 | 22 | 49 | 3 | 7 | 0 | 0.75 | 0.23 |
| 324 | 10414 | 5 | 129 | 2 | 9 | 0 | 1.18 | 0.03 |
| 333 | 8685 | 0 | 118 | 3 | 14 | 0 | 1.16 | -0.03 |
| 343 | 11511 | 7 | 92 | 0 | 6 | 0 | 0.81 | 0.06 |
| 344 | 12220 | 37 | 210 | 4 | 14 | 0 | 1.87 | 0.27 |
| 346 | 7915 | 2 | 174 | 0 | 3 | 0 | 2.19 | 0.03 |
| 347 | 17680 | 37 | 102 | 44 | 269 | 0 | -0.98 | -0.04 |
| 351 | 7678 | 7 | 185 | 3 | 0 | 0 | 2.46 | 0.05 |
|  |  |  |  |  |  |  |  |  |
| Average | 7630 | 8 | 146 | 3 | 10 | 0 | 1.94 | 0.05 |

Notes: (1) food and price are indicators of the food-versus-fuel narrative where GHG (including “greenhouse gas”), land, and ILUC are indicators of GHG narrative; (2) Narrative indicator 1 is the difference between food-versus-fuel related word count and GHG-related word count divided by total word count (((Food+Price)-(GHG+Land+ILUC) ) /Total); and (3) narrative indicator two is the difference between the incidences of “food” less the incidences of “GHG” divided by total word count ((Food-GHG) /Total).

Asterisks: * denotes short-run models, ** denotes if a study generates GHG-related results, and there are no studies that both report short-run model results and generate GHG results.

**S2. Data to support the hypothesis of a divergence in ethanol literature**

The presence of distinct bodies of literature is supported by online searches on key terms that show limited overlap.

Searches are conducted as follows to generate related indicators:

1. A few terms relating to the food-versus-fuel debate and the number of studies found is taken as an indicator of the size of the food-versus-fuel debate literature;
2. A few key words relating to the GHG debate and the number of studies found is taken as an indicator of the size of the GHG debate literature; and
3. All the terms above and the number of studies found is taken as an indicator of the intersection or overlap of these two bodies of literature.

Evidence of distinct bodies of literature would be as follows. First, online searches of studies would find few studies about food prices or food and fuel also discussed GHG emissions and indirect land use change. Second, few studies about GHG emissions or indirect land use change that also discuss food prices or food and fuel.

We do not make subjective judgments about what studies should or should not be included based on the lists generated. We report the number of hits from each search.

We anticipate a certain degree of apparent overlap in searches because studies might speak to broader matters in their introductory or concluding text than the contents strictly support. Studies might pique readers’ interest by discussing a wide set of research questions relating to ethanol or they might emphasize the importance of the results by drawing links to policy questions that might be tangentially related. Consequently, some food and fuel debate studies might include some comments about GHG emissions and some GHG studies will mention food price and the food-versus-fuel debate.

A small intersection of studies as compared to the numbers in each body of literature that use both terms would support our hypothesis that the bodies of literature are distinct. If the intersection of studies is large compared to the numbers of each body of literature, then our hypothesis is not supported.

Google Scholar searches for 2008 to 2017 on “food versus fuel” and “indirect land use change” separately produced 4,516 and 6,520 results, while a search on the intersection of these terms generated 591 results. The intersection accounts for 13% of the food-versus-fuel studies and 9% of the GHG studies. Overlap detected when searching for "food price" and “ethanol” (5,741) versus "greenhouse gas emissions" and “ethanol” (45,670) as compared to a combination requiring all these terms (1,970), is about one-third or less. We divide the period into two-year intervals to confirm that the overall results do not mask important differences at some particular point in time.

Table 5: Google Scholar

Note: searches conducted on March 19, 2018.

Web of Science searches were conducted on titles and topics, using basic settings and after specifying articles in English. Searches were undertaken for the ten-year period and for two five-year intervals. For topics, the basic search results give an intersection that is at most 39% the size of one of the bodies of literature. In most instances, the share is much smaller, often a tenth as large. The search focusing on articles in English also indicate a small intersection relative to the sizes of the two bodies of literature. Searches on titles results in few cases of overlap relative to the total numbers in each literature, or even none.

Table 6: Web of Science

Note: searches conducted on March 19, 2018, using both basic settings (columns to the left) and specifying with articles and English language specified (columns of data to the right).

Scopus searches are also on topic and title and cover the same time increments as Web of Science searches. Results from the Scopus search generate an intersection that is a bit less than 20% of a body of literature in one instance, but generally a very small number of overlapping studies.

Table 7: Scopus

Note: searches conducted on March 29, 2018.

Econ Lit searches were conducted for 2010-2018 focusing on entries in English. Of the searches conducted, at most 25% overlap is suggested and as little as 2%. The average is less than 10%.

Table 8: Econ Lit

Note: searches conducted on March 19, 2018.

Word frequency and correlation from 31 studies that are selected for analysis.

Using text mining package in R, we created a corpus of text from 31 studies after removing numbers, white space, punctuations, stopwords (e.g. “a”, “the”), words that appear less than 10 times and words with less than 3 characters. Variations of one keyword such as “emission” may include other stem words such as “emiss” and so on. After data cleaning, the original corpus contains 1414 keywords. To better show the frequency of words that are deemed to be most closely related to the two bodies of literature, we removed from the original corpus neutral-meaning words such as “also”, “amount”, “account”, and words with very low frequency. Figure 2 below shows the frequency of 42 most relevant words with “ethanol” and “price” accounting for 30% of the distribution.

Figure 2: Frequency of most relevant words with Pareto cumulative distribution (orange line)


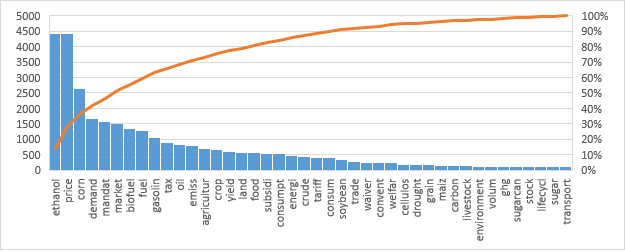


We found high correlation among GHG-related words such as “ghg”, “land”, “greenhouse”, “emission” or their stem word variations (e.g. “greenhous” or “emiss”), as shown in table 9. Correlations with “food” and “price” are less pronounced, likely due in part that “price” tends to appear in both sides of the literature. “Price” is one of the most frequently used words, second to “ethanol”, as shown in the above figure. It is also recognized that a study that focuses on analyzing a particular commodity, e.g. corn/maize, soybean, or cattle, may use terms that are specific to those commodities but still pointing to food price effects overall. A food-versus-fuel debate tends to point to the fact that high food prices might hurt consumers and households, therefore a number of other related words could have been used to make that point, as shown in table 10. As it is not clear how many thematic variants of food and food price might have been used in a study, we stick to general “food” and “price” word counts and use the relative frequencies of those words in a study, as well as compared to other studies, to account for how important these keywords are stressed in a food-versus-fuel literature.

Table 9: Correlation coefficients for GHG-related keywords

|  | ghg | land | greenhouse | emission |
| --- | --- | --- | --- | --- |
| ghg | 1 | 0.81 | 0.81 | 0.82 |
| land |  | 1 | 0.89 | 0.91 |
| greenhouse |  |  | 1 | 0.96 |
| emission |  |  |  | 1 |

Table 10: Correlation coefficients for food vs fuel-related keywords

|  | household | good | biofuel | energi | crop |  |
| --- | --- | --- | --- | --- | --- | --- |
| food | 0.75 | 0.69 | 0.68 | 0.68 | 0.67 |  |
|  |  |  |  |  |  |  |
|  | **suppli** | **demand** | **maiz** | **rose** | **futur** | **increas** |
| price | 0.71 | 0.7 | 0.68 | 0.62 | 0.6 | 0.67 |
|  |  |  |  |  |  |  |
|  | **maiz** | **egg** | **commod** | **soybean** | **feedstock** |  |
| spike | 0.76 | 0.67 | 0.64 | 0.62 | 0.6 |  |
|  |  |  |  |  |  |  |

**S3. Additional results**

Regressions presented here report the results of tests to see if results are sensitive, including to narrative indicator, random effects, clustering, or short-run price effects. We do not argue that sensitivity tests are all-inclusive, but we intend to show that the key coefficients remain statistically insignificant for at least this set of specifications.

This section also reports the results of supplemental analysis to detect whether control variables used in the regressions reported in the text are themselves a result of publication bias.

**S3.1. Alternative indicators of study narrative**

*Word count estimates*

Table 11. Word Count as a Function of Price Change Indicator.

|  | **Narrative indicator 2** | | |
| --- | --- | --- | --- |
|  | (1) | (2) | (3) |
| Model 1 | | | |
| Average price change | 0.061 | 0.061 | 0.073 |
|  | (0.060) | (0.061) | (0.112) |
| Journal publication (Yes=1, No=0) |  | -0.041 | -0.031 |
|  |  | (0.083) | (0.119) |
| Average price change x Journal publication |  |  | -0.017 |
|  |  |  | (0.134) |
| Constant | 0.038 | 0.063 | 0.056 |
|  | (0.055) | (0.076) | (0.097) |
|  |  |  |  |
| R^2^ | 0.028 | 0.035 | 0.035 |
| Adjusted R^2^ | 0.001 | -0.02 | -0.05 |
| Model 2 |  |  |  |
| Semi-elasticity price change | -0.01 | -0.013 | -0.003 |
|  | (0.013) | (0.014) | (0.017) |
| Journal publication (Yes=1, No=0) | | -0.065 | 0.06 |
|  |  | (0.090) | (0.164) |
| Semi-elasticity price change x Journal publication | |  | -0.027 |
|  |  |  | (0.030) |
| Constant | 0.123 | 0.179 | 0.122 |
|  | (0.074) | (0.107) | (0.124) |
|  |  |  |  |
| R^2^ | 0.015 | 0.03 | 0.054 |
| Adjusted R^2^ | -0.013 | -0.027 | -0.032 |
| Observations | 37 | 37 | 37 |

Notes: * p<.05   ** p<.01   *** p<.001. Narrative indicator 2=Share of food vs. GHG words in total word count.

*Corn price effect estimates*

Table 12. Word Count as a Function of Maximum Price Change Indicator.

|  | **Narrative indicator 2** | | |
| --- | --- | --- | --- |
|  | (1) | (2) | (3) |
| Model 1 | | | |
| Maximum corn price change | 0.025 | 0.026 | 0.049 |
|  | (0.038) | (0.039) | (0.090) |
| Journal publication (Yes=1, No=0) |  | -0.044 | -0.019 |
|  |  | (0.084) | (0.123) |
| Maximum corn price change x Journal publication |  |  | -0.028 |
|  |  |  | (0.100) |
| Constant | 0.053 | 0.079 | 0.059 |
|  | (0.054) | (0.074) | (0.103) |
|  |  |  |  |
| R^2^ | 0.012 | 0.019 | 0.022 |
| Adjusted R^2^ | -0.016 | -0.037 | -0.065 |
| Model 2 |  |  |  |
| Maximum semi-elasticity corn price change | -0.006 | -0.009 | -0.005 |
|  | (0.009) | (0.009) | (0.014) |
| Journal publication (Yes=1, No=0) |  | -0.068 | -0.023 |
|  |  | (0.090) | (0.157) |
| Maximum semi-elasticity corn price change e x Journal publication |  |  | -0.007 |
|  |  |  | (0.019) |
| Constant | 0.118 | 0.175 | 0.145 |
|  | (0.068) | (0.102) | (0.134) |
|  |  |  |  |
| R^2^ | 0.016 | 0.032 | 0.036 |
| Adjusted R^2^ | -0.012 | -0.025 | -0.052 |
| Observations | 37 | 37 | 37 |

Notes: * p<.05   ** p<.01   *** p<.001. Narrative indicator 2=Share of food vs. GHG words in total word count.

Table 13. Corn Price Change as a Function of Narrative (indicator 2).

|  | **Corn price change per billion gallon increase in ethanol** | | | | |
| --- | --- | --- | --- | --- | --- |
|  |  | (1) | (2) | (3) | (4) |
| (Intercept) |  | 0.606*** | 0.227 | -0.184 | -0.247 |
|  |  | (0.084) | (0.133) | (0.177) | (0.179) |
| Narrative indicator 2 |  | -0.336 | 0.217 | 0.535 | 1.374* |
|  |  | (0.414) | (0.460) | (0.294) | (0.670) |
| Journal publication (1=yes, 0=no) |  |  | -0.329** | -0.086 | 0.026 |
|  |  |  | (0.117) | (0.154) | (0.170) |
| Initial level of corn ethanol (b.g.) |  |  | 0.016** | 0.018** | 0.018** |
|  |  |  | (0.006) | (0.006) | (0.006) |
| Corn ethanol change (b.g.) |  |  | 0.093*** | 0.121*** | 0.117*** |
|  |  |  | (0.009) | (0.015) | (0.015) |
| Corn ethanol change squared (b.g.) |  |  |  | -0.001* | -0.001 |
|  |  |  |  | -0.0005 | -0.0005 |
| Decrease scenario (1=yes, 0=no) |  |  | -0.032 | 0.051 | 0.025 |
|  |  |  | (0.114) | (0.123) | (0.122) |
| Short run model (1=yes, 0=no) |  |  |  | 0.475** | 0.436** |
|  |  |  |  | (0.161) | (0.158) |
| Narrative indicator 2 x Journal Publication |  |  |  |  | -1.054 |
|  |  |  |  |  | (0.749) |
|  |  |  |  |  |  |
| Number of groups |  | 38 | 38 | 38 | 38 |
| Observations |  | 221 | 221 | 221 | 221 |
| Akaike information criterion (AIC) |  | 382.113 | 301.36 | 295.72 | 295.786 |

Notes: * p<.05   ** p<.01   *** p<.001. Narrative indicator 2=Share of food vs. GHG words in total word count.

Table 14. Corn price change (%) per billion gallon increase in corn ethanol as a Function of Narrative (indicator 2).

|  | **Corn price change (%) per billion gallon increase in ethanol** | | | | |
| --- | --- | --- | --- | --- | --- |
|  |  | (1) | (2) | (3) | (4) |
| (Intercept) |  | 5.016*** | 9.571*** | 7.896*** | 7.931*** |
|  |  | (0.525) | (0.899) | (1.041) | (1.102) |
| Narrative indicator 2 |  | -2.209 | -1.726 | -1.547 | -1.946 |
|  |  | (2.070) | (1.746) | (1.617) | (4.287) |
| Journal publication (1=yes, 0=no) |  |  | -2.337** | -0.931 | -0.979 |
|  |  |  | (0.876) | (0.948) | (1.061) |
| Initial level of corn ethanol (b.g.) |  |  | -0.142*** | -0.139*** | -0.139*** |
|  |  |  | (0.033) | (0.033) | (0.033) |
| Corn ethanol change (b.g.) |  |  | -0.157*** | -0.151*** | -0.151*** |
|  |  |  | (0.047) | (0.045) | (0.045) |
| Decrease scenario (1=yes, 0=no) |  |  | -2.131** | -2.044** | -2.034** |
|  |  |  | (0.797) | (0.743) | (0.751) |
| Short run model (1=yes, 0=no) |  |  |  | 2.733** | 2.748** |
|  |  |  |  | (0.993) | (1.001) |
| Narrative indicator 2 x Journal Publication |  |  |  |  | 0.467 |
|  |  |  |  |  | (4.590) |
|  |  |  |  |  |  |
| Number of groups |  | 37 | 37 | 37 | 37 |
| Observations |  | 220 | 220 | 220 | 220 |
| Akaike information criterion (AIC) |  | 1,096.10 | 1,068.77 | 1,063.98 | 1,065.97 |

Notes: * p<.05   ** p<.01   *** p<.001. Narrative indicator 2=Share of food vs. GHG words in total word count.

Table 15. GHG-related Study Binary Variable as a Function of Average Corn Price Change

|  | **If the study published GHG-related results (1=yes, 0= no)** | | |
| --- | --- | --- | --- |
|  | (1) | (2) | (3) |
| Model 1 | | | |
| Average price change | 0.374 | 0.363 | -7.488 |
|  | (0.762) | (0.761) | (9.203) |
| Journal publication (Yes=1, No=0) |  | 0.609 | -2.066 |
|  |  | (1.211) | (2.113) |
| Average price change x Journal publication |  |  | 8.314 |
|  |  |  | (9.245) |
| Constant | -2.403** | -2.812* | -0.533 |
|  | (0.790) | (1.184) | (1.845) |
|  |  |  |  |
| Akaike Inf. Crit. | 29.34 | 31.068 | 30.722 |
| Model 2 |  |  |  |
| Semi-elasticity price change | 0.078 | 0.136 | -1.641 |
|  | (0.150) | (0.173) | (1.542) |
| Journal publication (Yes=1, No=0) | | 1.048 | -7.948 |
|  |  | (1.374) | (4.996) |
| Semi-elasticity price change x Journal publication | |  | 2.217 |
|  |  |  | (1.575) |
| Constant | -2.498** | -3.506* | 3.241 |
|  | (0.957) | (1.738) | (4.596) |
|  |  |  |  |
| Akaike Inf. Crit. | 29.098 | 30.433 | 26.579 |
| Observations | 38 | 38 | 38 |

Notes: * p<.05   ** p<.01   *** p<.001. Semi-elasticity price change is the percent change of corn price per 1 billion gallon increase in corn starch ethanol.

Table 16. Corn Price Change as a Function of GHG-related Study Binary Variable

|  |  | **Corn price change per billion gallon increase in ethanol** | | | | |
| --- | --- | --- | --- | --- | --- | --- |
|  |  |  | (1) | (2) | (3) | (4) |
| (Intercept) |  |  | 0.635*** | 0.228 | -0.119 | -0.101 |
|  |  |  | (0.109) | (0.153) | (0.177) | (0.176) |
| GHG-related study (yes=1, no=0) |  |  | 0.173 | 0.196 | 0.291 | -0.122 |
|  |  |  | (0.342) | (0.238) | (0.215) | (0.511) |
| Journal publication (1=yes, 0=no) |  |  |  | -0.302* | -0.076 | -0.107 |
|  |  |  |  | (0.151) | (0.155) | (0.156) |
| Initial level of corn ethanol (b.g.) |  |  |  | 0.016** | 0.016** | 0.016** |
|  |  |  |  | (0.006) | (0.006) | (0.006) |
| Corn ethanol change (b.g.) |  |  |  | 0.090*** | 0.112*** | 0.112*** |
|  |  |  |  | (0.008) | (0.015) | (0.015) |
| Corn ethanol change squared (b.g.) |  |  |  |  | -0.001 | -0.001 |
|  |  |  |  |  | (0.001) | (0.001) |
| Decrease scenario (1=yes, 0=no) |  |  |  | -0.013 | 0.049 | 0.069 |
|  |  |  |  | (0.138) | (0.126) | (0.126) |
| Short run model (1=yes, 0=no) |  |  |  |  | 0.453** | 0.431** |
|  |  |  |  |  | (0.165) | (0.163) |
| Narrative indicator 1 x Journal Publication |  |  |  |  |  | 0.503 |
|  |  |  |  |  |  | (0.562) |
|  |  |  |  |  |  |  |
| Number of groups |  |  | 38 | 38 | 38 | 38 |
| Observations |  |  | 221 | 221 | 221 | 221 |
| Akaike information criterion (AIC) |  |  | 386.478 | 299.383 | 294.584 | 295.798 |

Notes: * p<.05   ** p<.01   *** p<.001.

**S3.2. Random effects**

Table 17. Supplemental Experiments for Equations 4 and 5, with Random Intercept and Random Slope for the Word Count Variable.

|  | *Dependent variable:* | | | |
| --- | --- | --- | --- | --- |
|  |  | | | |
|  | Corn ethanol change (b.g.) | | Initial level of corn ethanol (b.g.) | |
|  | (1) | (2) | (3) | (4) |
|  | | | | |
| Narrative indicator 1 | 0.872 | 1.273 | -0.553 | -0.808 |
|  | (0.623) | (2.951) | (0.630) | (2.303) |
|  |  |  |  |  |
| Journal publication (1=yes, 0=no) | 4.432^*^ | 5.197 | -2.635 | -3.179 |
|  | (2.173) | (5.924) | (1.836) | (5.056) |
|  |  |  |  |  |
| Narrative indicator 1*Journal publication |  | -0.419 |  | 0.276 |
|  |  | (3.019) |  | (2.394) |
|  |  |  |  |  |
| Constant | 0.316 | -0.447 | 12.459*** | 12.985^**^ |
|  | (2.060) | (5.873) | (1.870) | (4.925) |
|  |  |  |  |  |
| Observations | 221 | 221 | 221 | 221 |
| Log Likelihood | -596.552 | -596.543 | -693.298 | -693.291 |
| Akaike Inf. Crit. | 1,207.11 | 1,209.09 | 1,400.596 | 1,402.58 |
|  | | | | |
| Notes: * p<.05   ** p<.01   *** p<.001. Narrative indicator 1=Share of food-related vs. GHG-related words in total word count. | | | | |

Table 18. Supplemental Experiments for Equations 4 and 5, with Random Intercept Only.

|  | *Dependent variable:* | | | |
| --- | --- | --- | --- | --- |
|  |  | | | |
|  | Corn ethanol change (b.g.) | | Initial level of corn ethanol (b.g.) | |
|  | (1) | (2) | (3) | (4) |
|  | | | | |
| Narrative indicator 1 | 0.099 | 0.951 | -0.375 | -0.664 |
|  | (0.793) | (2.678) | (0.714) | (2.129) |
|  |  |  |  |  |
| Journal publication (1=yes, 0=no) | 4.380* | 6.29 | -2.34 | -3.004 |
|  | (2.113) | (6.105) | (1.761) | (4.939) |
|  |  |  |  |  |
| Narrative indicator 1*Journal publication |  | -0.934 |  | 0.325 |
|  |  | (2.803) |  | (2.260) |
|  |  |  |  |  |
| Constant | 2.034 | 0.219 | 12.128*** | 12.748** |
|  | (2.293) | (5.907) | (1.981) | (4.741) |
|  |  |  |  |  |
| Observations | 221 | 221 | 221 | 221 |
| Log Likelihood | -599.304 | -599.249 | -693.74 | -693.728 |
| Akaike Inf. Crit. | 1,208.61 | 1,210.50 | 1,397.48 | 1,399.46 |
|  | | | | |
| Notes: * p<.05   ** p<.01   *** p<.001. Narrative indicator 1=Share of food-related vs. GHG-related words in total word count. | | | | |

Table 19. Supplemental Experiments for Equations 4 and 5, with Random Intercept Only and with a Short-Run Dummy.

|  | *Dependent variable:* | | | |
| --- | --- | --- | --- | --- |
|  |  | | | |
|  | Corn ethanol change (b.g.) | | Initial level of corn ethanol (b.g.) | |
|  | (1) | (2) | (3) | (4) |
|  | | | | |
| Narrative indicator 1 | 0.064 | 0.628 | -0.367 | -0.66 |
|  | (0.796) | (2.896) | (0.724) | (2.412) |
|  |  |  |  |  |
| Journal publication (1=yes, 0=no) | 3.776 | 5.149 | -2.258 | -2.991 |
|  | (2.608) | (7.253) | (2.136) | (6.148) |
|  |  |  |  |  |
| Short run study (yes=1, no=0) | -1.083 | -0.861 | 0.148 | 0.009 |
|  | (2.757) | (2.966) | (2.185) | (2.443) |
|  |  |  |  |  |
| Narrative indicator 1*Journal publication |  | -0.611 |  | 0.321 |
|  |  | (3.012) |  | (2.526) |
|  |  |  |  |  |
| Constant | 2.729 | 1.4 | 12.027*** | 12.734* |
|  | (2.893) | (7.167) | (2.485) | (6.096) |
|  |  |  |  |  |
| Observations | 221 | 221 | 221 | 221 |
| Log Likelihood | -599.227 | -599.207 | -693.74 | -693.728 |
| Akaike Inf. Crit. | 1,210.46 | 1,212.41 | 1,399.47 | 1,401.46 |
|  | | | | |
| Notes: * p<.05   ** p<.01   *** p<.001. Narrative indicator 1=Share of food-related vs. GHG-related words in total word count. | | | | |

Table 20. Supplemental Experiments for Equations 4 and 5 (1 of 2), with Random Intercept Only and with Model Type Dummies.

|  | *Dependent variable:* | | | | | | | |  |
| --- | --- | --- | --- | --- | --- | --- | --- | --- | --- |
|  |  | | | | | | | |  |
|  | Corn ethanol change (b.g.) | | | | Initial level of corn ethanol (b.g.) | | | |  |
|  | (1) | | (2) | | (3) | | (4) | |  |
|  | | | | | | | | |  |
| Narrative indicator 1 | 0.91 | | -0.939 | | -0.68 | | -0.803 | |  |
|  | (0.785) | | (2.731) | | (0.766) | | (2.383) | |  |
|  |  | |  | |  | |  | |  |
| Journal publication | 4.644 | | 0.142 | | -2.285 | | -2.595 | |  |
|  | (2.504) | | (6.837) | | (2.153) | | (6.081) | |  |
| Model type (GTAP is omitted) |  | |  | |  | |  | |  |
| *2-GE_other* | 5.211 | | 5.44 | | -0.377 | | -0.36 | |  |
|  | (4.815) | | (4.798) | | (4.238) | | (4.253) | |  |
|  |  | |  | |  | |  | |  |
| *3-FAPRI-ISU* | 3.163 | | 3.88 | | 3.156 | | 3.205 | |  |
|  | (4.620) | | (4.707) | | (4.281) | | (4.377) | |  |
|  |  | |  | |  | |  | |  |
| *4-FAPRI-MU* | -3.494 | | -3.617 | | 10.027 | | 10.017 | |  |
|  | (5.507) | | (5.479) | | (5.491) | | (5.496) | |  |
|  |  | |  | |  | |  | |  |
| *5-ShortRunPE* | -1.634 | | -2.245 | | 2.629 | | 2.577 | |  |
|  | (4.591) | | (4.644) | | (4.015) | | (4.125) | |  |
|  |  | |  | |  | |  | |  |
| *6-PE_other* | -2.807 | | -2.914 | | 2.612 | | 2.605 | |  |
|  | (3.978) | | (3.957) | | (3.579) | | (3.582) | |  |
|  |  | |  | |  | |  | |  |
| Narrative indicator 1*Journal publication | | | 2.079 | |  | | 0.141 | |  |
|  |  | | (2.940) | |  | | (2.577) | |  |
|  |  | |  | |  | |  | |  |
| Constant | 1.622 | | 5.843 | | 10.121^*^ | | 10.411 | |  |
|  | (4.775) | | (7.626) | | (4.256) | | (6.803) | |  |
|  |  | |  | |  | |  | |  |
|  | | | | | | | | |  |
| Observations | 221 | | 221 | | 221 | | 221 | |  |
| Log Likelihood | -595.471 | | -595.223 | | -691.451 | | -691.449 | |  |
| Akaike Inf. Crit. | 1,210.94 | | 1,212.45 | | 1,402.90 | | 1,404.90 | |  |
|  | | | | | | | | |  |
| *Note:* | ^*^p<0.05; ^**^p<0.01; ^***^p<0.001 | | | | | | | |  |
|  | | *Dependent variable:* | | | | | | | |
|  | |  | | | | | | | |
|  | | Corn ethanol change (b.g.) | | | | Initial level of corn ethanol (b.g.) | | | |
|  | | (1) | | (2) | | (3) | | (4) | |
|  | | | | | | | | | |
| Narrative indicator 1 | | 0.91 | | -0.939 | | -0.68 | | -0.803 | |
|  | | (0.785) | | (2.731) | | (0.766) | | (2.383) | |
|  | |  | |  | |  | |  | |
| Journal publication | | 4.644 | | 0.142 | | -2.285 | | -2.595 | |
|  | | (2.504) | | (6.837) | | (2.153) | | (6.081) | |
| Model type (GTAP is omitted) | |  | |  | |  | |  | |
| *2-GE_other* | | 5.211 | | 5.44 | | -0.377 | | -0.36 | |
|  | | (4.815) | | (4.798) | | (4.238) | | (4.253) | |
|  | |  | |  | |  | |  | |
| *3-FAPRI-ISU* | | 3.163 | | 3.88 | | 3.156 | | 3.205 | |
|  | | (4.620) | | (4.707) | | (4.281) | | (4.377) | |
|  | |  | |  | |  | |  | |
| *4-FAPRI-MU* | | -3.494 | | -3.617 | | 10.027 | | 10.017 | |
|  | | (5.507) | | (5.479) | | (5.491) | | (5.496) | |
|  | |  | |  | |  | |  | |
| *5-ShortRunPE* | | -1.634 | | -2.245 | | 2.629 | | 2.577 | |
|  | | (4.591) | | (4.644) | | (4.015) | | (4.125) | |
|  | |  | |  | |  | |  | |
| *6-PE_other* | | -2.807 | | -2.914 | | 2.612 | | 2.605 | |
|  | | (3.978) | | (3.957) | | (3.579) | | (3.582) | |
|  | |  | |  | |  | |  | |
| Narrative indicator 1*Journal publication | | | | 2.079 | |  | | 0.141 | |
|  | |  | | (2.940) | |  | | (2.577) | |
|  | |  | |  | |  | |  | |
| Constant | | 1.622 | | 5.843 | | 10.121^*^ | | 10.411 | |
|  | | (4.775) | | (7.626) | | (4.256) | | (6.803) | |
|  | |  | |  | |  | |  | |
|  | | | | | | | | | |
| Observations | | 221 | | 221 | | 221 | | 221 | |
| Log Likelihood | | -595.471 | | -595.223 | | -691.451 | | -691.449 | |
| Akaike Inf. Crit. | | 1,210.94 | | 1,212.45 | | 1,402.90 | | 1,404.90 | |
|  | | | | | | | | | |
| Notes: * p<.05   ** p<.01   *** p<.001. Narrative indicator 1=Share of food-related vs. GHG-related words in total word count. | | | | | | | | | |

**S3.3. Alternative measures of price effects**

Table 21. Supplemental Experiments for Equations 4 and 5, Using only the Average Value for Each Study.

|  | *Dependent variable:* | | | |
| --- | --- | --- | --- | --- |
|  |  | | | |
|  | Corn ethanol change (b.g.) | | Initial level of corn ethanol (b.g.) | |
|  | (1) | (2) | (3) | (4) |
|  | | | | |
| Narrative indicator 1 | 0.035 | 0.912 | -0.606 | -1.08 |
|  | (0.818) | (2.915) | (0.698) | (2.491) |
|  |  |  |  |  |
| Journal publication (1=yes, 0=no) | 4.300 | 6.253 | (2.209) | (3.264) |
|  | -2.241 | -6.62 | -1.913 | -5.658 |
|  |  |  |  |  |
| Narrative indicator 1*Journal publication |  | -0.955 |  | 0.516 |
|  |  | (3.040) |  | (2.599) |
|  |  |  |  |  |
| Constant | 2.172 | 0.307 | 12.292^***^ | 13.300^*^ |
|  | -2.404 | -6.419 | -2.053 | -5.486 |
|  |  |  |  |  |
|  | | | | |
| Observations | 38 | 38 | 38 | 38 |
| R^2^ | 0.106 | 0.109 | 0.043 | 0.044 |
| Adjusted R^2^ | 0.055 | 0.03 | -0.012 | -0.04 |
|  | | | | |

Notes: * p<.05   ** p<.01   *** p<.001. Narrative indicator 1=Share of food-related vs. GHG-related words in total word count.

**S3.4. Supplemental analysis to test if bias (narrative) causes control variables**

Table 22. Supplemental Experiments for Equations 4 and 5.

|  | *Dependent variable:* | |
| --- | --- | --- |
|  |  | |
|  | Corn price change (%) per billion gallon increase in corn ethanol | Corn price change ($/bu) |
|  | (1) | (2) |
|  | | |
| Narrative indicator 1 | -0.13 | -0.005 |
|  | (0.349) | (0.054) |
|  |  |  |
| Journal publication | -1.041 | -0.091 |
|  | (0.938) | (0.127) |
|  |  |  |
| Initial level of corn ethanol (b.g.) | -0.133^***^ | 0.016^**^ |
|  | (0.033) | (0.006) |
|  |  |  |
| Corn ethanol change (b.g.) | -0.178^***^ | 0.127^***^ |
|  | (0.046) | (0.015) |
|  |  |  |
| Corn ethanol change squared (b.g.) |  | -0.001^**^ |
|  |  | (0.001) |
|  |  |  |
| Decrease scenario (1=yes, 0=no) | -1.818^*^ | 0.14 |
|  | (0.708) | (0.102) |
| Model type (GTAP is omitted) |  |  |
| *2-GE_other* | 5.174^**^ | 1.119^***^ |
|  | (1.841) | (0.281) |
|  |  |  |
| *3-FAPRI-ISU* | 2.04 | 0.379 |
|  | (1.859) | (0.279) |
|  |  |  |
| *4-FAPRI-MU* | 2.389 | 0.478 |
|  | (2.448) | (0.387) |
|  |  |  |
| *5-ShortRunPE* | 5.379^**^ | 1.052^***^ |
|  | (1.727) | (0.252) |
|  |  |  |
| *6-PE_other* | 3.194^*^ | 0.734^**^ |
|  | (1.557) | (0.231) |
|  |  |  |
| Constant | 5.173^**^ | -0.798^**^ |
|  | (1.892) | (0.289) |
|  |  |  |
|  | | |
| Observations | 220 | 221 |
| Log Likelihood | -518.223 | -130.389 |
| Akaike Inf. Crit. | 1,062.45 | 288.779 |
|  | | |
| Notes: * p<.05   ** p<.01   *** p<.001. Narrative indicator 1=Share of food-related vs. GHG-related words in total word count. | | |

Figure 4. Experiment Relating to Equation 2 - Model Coefficient Estimates from Corn Price Change Regression versus Word Count.

Notes: the x-axis is the estimated coefficient associated with model in the mixed effects regression or price effects; and the y-axis is the relative word counts of food-versus-fuel and GHG literatures (narrative indicator 1) for the studies that use the model. A relationship between word count and model effects on the size of ethanol-shock price effect would be evidence of publication bias.

**S3.5. Clustering by model**

Table 23. Relative Price Change as a Function of Narrative Indicator 1.

|  | *Dependent variable:* | | |
| --- | --- | --- | --- |
|  |  | | |
|  | Corn price change (%) per billion gallon increase in ethanol | | |
|  | (1) | (2) | (3) |
| Narrative indicator 1 | -0.376 | -0.207 | 0.062 |
|  | (0.302) | (0.292) | (0.765) |
|  |  |  |  |
| Journal publication | -1.841^**^ | -0.821 | -0.212 |
|  | (0.653) | (0.732) | (1.779) |
|  |  |  |  |
| Initial level of corn ethanol (b.g.) | -0.132^***^ | -0.126^***^ | -0.125^***^ |
|  | (0.034) | (0.034) | (0.034) |
|  |  |  |  |
| Corn ethanol change (b.g.) | -0.185^***^ | -0.185^***^ | -0.185^***^ |
|  | (0.047) | (0.045) | (0.045) |
|  |  |  |  |
| Decrease scenario (1=yes, 0=no) | -2.128^**^ | -2.305^***^ | -2.352^***^ |
|  | (0.741) | (0.687) | (0.692) |
|  |  |  |  |
| Short run study (yes=1, no=0) |  | 2.401^**^ | 2.469^**^ |
|  |  | (0.852) | (0.868) |
|  |  |  |  |
| Narrative indicator 1*Journal publication |  |  | -0.31 |
|  |  |  | (0.817) |
|  |  |  |  |
| Constant | 9.875^***^ | 8.514^***^ | 7.941^***^ |
|  | (0.943) | (1.040) | (1.851) |
|  |  |  |  |
|  | | | |
| Observations | 220 | 220 | 220 |
| Log Likelihood | -530.843 | -527.162 | -527.091 |
| Akaike Inf. Crit. | 1,077.69 | 1,072.33 | 1,074.18 |
|  | | | |
| Notes: * p<.05   ** p<.01   *** p<.001. Narrative indicator 1=Share of food-related vs. GHG-related words in total word count. | | | |

Table 24. Relative Price Change as a Function of GHG binary variable.

|  | *Dependent variable:* | | |
| --- | --- | --- | --- |
|  |  | | |
|  | Corn price change (%) per billion gallon increase in ethanol | | |
|  | (1) | (2) | (3) |
|  | | | |
| GHG-related study | 1.891 | 2.165^*^ | -1.275 |
|  | (1.199) | (1.071) | (2.787) |
|  |  |  |  |
| Journal publication | -1.762^**^ | -0.787 | -0.949 |
|  | (0.633) | (0.664) | (0.662) |
|  |  |  |  |
| Initial level of corn ethanol (b.g.) | -0.132^***^ | -0.126^***^ | -0.125^***^ |
|  | (0.034) | (0.034) | (0.033) |
|  |  |  |  |
| Corn ethanol change (b.g.) | -0.186^***^ | -0.183^***^ | -0.183^***^ |
|  | (0.046) | (0.044) | (0.044) |
|  |  |  |  |
| Decrease scenario (1=yes, 0=no) | -2.241^**^ | -2.288^***^ | -2.197^***^ |
|  | (0.710) | (0.635) | (0.630) |
|  |  |  |  |
| Short run study (yes=1, no=0) |  | 2.622^***^ | 2.500^**^ |
|  |  | (0.781) | (0.768) |
|  |  |  |  |
| Narrative indicator 1*Journal publication |  |  | 4.005 |
|  |  |  | (3.008) |
|  |  |  |  |
| Constant | 9.083^***^ | 7.908^***^ | 8.004^***^ |
|  | (0.799) | (0.837) | (0.827) |
|  |  |  |  |
|  | | | |
| Observations | 220 | 220 | 220 |
| Log Likelihood | -530.394 | -525.46 | -524.584 |
| Akaike Inf. Crit. | 1,076.79 | 1,068.92 | 1,069.17 |
|  | | | |

**S3.6. Exclude short-run studies**

Table 25. Relative Price Change as a Function of Narrative Indicator 1.

|  | *Dependent variable:* | | |
| --- | --- | --- | --- |
|  |  | | |
|  | Corn price change (%) per billion gallon increase in ethanol | | |
|  | (1) | (2) | (3) |
|  | | | |
| Narrative indicator 1 | -0.418 | -0.082 | -0.364 |
|  | (0.334) | (0.397) | (1.130) |
|  |  |  |  |
| Journal publication |  | -0.43 | -1.295 |
|  |  | (0.806) | (3.234) |
|  |  |  |  |
| Initial level of corn ethanol (b.g.) |  | -0.138^***^ | -0.138^***^ |
|  |  | (0.035) | (0.035) |
|  |  |  |  |
| Corn ethanol change (b.g.) |  | -0.161^***^ | -0.161^***^ |
|  |  | (0.046) | (0.046) |
|  |  |  |  |
| Decrease scenario (1=yes, 0=no) |  | -1.367 | -1.348 |
|  |  | (0.841) | (0.842) |
|  |  |  |  |
| Constant |  |  | 0.333 |
|  |  |  | (1.177) |
|  |  |  |  |
| Constant | 4.881^***^ | 7.609^***^ | 8.385^**^ |
|  | (0.806) | (1.261) | (3.144) |
|  |  |  |  |
|  | | | |
| Observations | 171 | 171 | 171 |
| Log Likelihood | -422.361 | -410.789 | -410.753 |
| Akaike Inf. Crit. | 856.722 | 841.578 | 843.506 |

Notes: * p<.05   ** p<.01   *** p<.001. Narrative indicator 1=Share of food-related vs. GHG-related words in total word count.

Table 26. Corn Price Change as a Function of GHG binary variable.

|  | *Dependent variable:* | | |
| --- | --- | --- | --- |
|  |  | | |
|  | Corn price change ($/bu.) | | |
|  | (1) | (2) | (3) |
|  | | | |
| GHG-related study | 0.238 | 0.232 | -0.167 |
|  | (0.350) | (0.204) | (0.472) |
|  |  |  |  |
| Journal publication |  | -0.185 | -0.228 |
|  |  | (0.181) | (0.183) |
|  |  |  |  |
| Initial level of corn ethanol (b.g.) |  | 0.014^**^ | 0.014^**^ |
|  |  | (0.005) | (0.005) |
|  |  |  |  |
| Corn ethanol change (b.g.) |  | 0.078^***^ | 0.078^***^ |
|  |  | (0.007) | (0.007) |
|  |  |  |  |
| Decrease scenario (1=yes, 0=no) |  | -0.193 | -0.175 |
|  |  | (0.146) | (0.146) |
|  |  |  |  |
| Short run study (yes=1, no=0) |  |  | 0.491 |
|  |  |  | (0.523) |
|  |  |  |  |
| GHG-related study indicator*Journal publication | 0.567^***^ | 0.223 | 0.249 |
|  | (0.130) | (0.197) | (0.195) |
|  |  |  |  |
|  | | | |
| Observations | 172 | 172 | 172 |
| Log Likelihood | -128.868 | -85.359 | -84.926 |
| Akaike Inf. Crit. | 265.737 | 186.718 | 187.852 |
|  | | | |

Table 27. Narrative as a Function of Price Change (Average Value).

|  | **Narrative as a Function of Price Change (Average Value).** | |
| --- | --- | --- |
|  | (1) | (2) |
| Model 1 | | |
| Average price change | 0.043 | 0.656 |
|  | (0.155) | (0.362) |
| Journal publication (Yes=1, No=0) | -1.070*** | -0.531 |
|  | (0.152) | (0.326) |
| Average price change x Journal publication |  | -0.747 |
|  |  | (0.400) |
| Constant | 2.777*** | 2.346*** |
|  | (0.169) | (0.285) |
|  |  |  |
| Adjusted R^2^ | 0.219 | 0.23 |
| Model 2 |  |  |
| Semi-elasticity price change | 1.407*** | -3.101 |
|  | (0.333) | (1.773) |
| Journal publication (Yes=1, No=0) | -0.709 | -18.939** |
|  | (1.208) | (5.927) |
| Semi-elasticity price change x Journal publication | | 4.825** |
|  |  | (1.824) |
| Constant | -9.888*** | 6.42 |
|  | (1.918) | (5.306) |
|  |  |  |
| Akaike Inf. Crit. | 50.26 | 43.833 |
| Observations | 38 | 38 |

Notes: * p<.05   ** p<.01   *** p<.001. Narrative indicator 1=Share of food-related vs. GHG-related words in total word count.

**S4. List of 38 studies**

1. Babcock, B. (2008). Distributional implications of US ethanol policy. *Applied Economic Perspectives and Policy*, *30*(3), 533–542.
2. Babcock, B. (2011). *The Impact of US Biofuel Policies on Agricultural Price Levels and Volatility* (Issue Paper No. 35). International Centre for Trade and Sustainable Development (ICTSD).
3. Babcock, B. (2012). *Updated assessment of the drought’s impacts on crop prices and biofuel production* [CARD Policy Brief]. Center for Agricultural and Rural Development, Iowa State University. <http://www.card.iastate.edu/publications/DBS/PDFFiles/12pb8.pdf>
4. Babcock, B., Barr, K. J., Carriquiry, M. A., & others. (2010). *Costs and benefits to taxpayers, consumers, and producers from US ethanol policies*. Center for Agricultural and Rural Development, Iowa State University Ames, IA. <http://www.agmanager.info/about/contributors/Presentations/Langemeier/Babcock_2010.pdf>
5. Babcock, B., & Fabiosa, J. (2011). *The Impact of Ethanol and Ethanol Subsidies on Corn Prices: Revisiting History.* [CARD Policy Brief 11-PB 5]. Center for Agricultural and Rural Development, Iowa State University.
6. Babcock, B., & Zhou, W. (2013). *Impact on Corn Prices from Reduced Biofuel Mandates*. Center for Agricultural and Rural Development , Iowa State University. <http://www.card.iastate.edu/publications/synopsis.aspx?id=1215>
7. Bento, A. M., & Klotz, R. (2014). Climate policy decisions require policy-based lifecycle analysis. *Environmental Science and Technology*, *48*(10), 5379–5387. Scopus. <https://doi.org/10.1021/es405164g>
8. Bento, A. M., Klotz, R., & Landry, J. R. (2015). Are there Carbon Savings from US Biofuel Policies? The Critical Importance of Accounting for Leakage in Land and Fuel Markets. *The Energy Journal*, *36*(3). <https://doi.org/10.5547/01956574.36.3.aben>
9. Chen, X., & Khanna, M. (2012). Food vs. Fuel: The Effect of Biofuel Policies. *American Journal of Agricultural Economics*, aas039. <https://doi.org/10.1093/ajae/aas039>
10. Chen, X., & Khanna, M. (2018). Effect of corn ethanol production on Conservation Reserve Program acres in the US. *Applied Energy*, *225*, 124–134. <https://doi.org/10.1016/j.apenergy.2018.04.104>
11. Cui, J., Lapan, H., Moschini, G., & Cooper, J. (2011). Welfare Impacts of Alternative Biofuel and Energy Policies. *American Journal of Agricultural Economics*, aar053. <https://doi.org/10.1093/ajae/aar053>
12. de Gorter, H., & Just, D. R. (2008a). The economics of the US ethanol import tariff with a blend mandate and tax credit. *Journal of Agricultural & Food Industrial Organization*, *6*(2). <https://doi.org/10.2202/1542-0485.1239>
13. de Gorter, H., & Just, D. R. (2008b). *The law of unintended consequences: How the US biofuel tax credit with a mandate subsidizes oil consumption and has no impact on ethanol consumption* (WP 2007-20). Cornell University, Department of Applied Economics and Management. <http://papers.ssrn.com/sol3/papers.cfm?abstract_id=1024525>
14. Dhoubhadel, S. P., Azzam, A. M., & Stockton, M. C. (2015). The Impact of Biofuels Policy and Drought on the U.S. Grain and Livestock Markets. *Journal of Agricultural and Applied Economics*, *47*(01), 77–103. <https://doi.org/10.1017/aae.2014.6>
15. Elobeid, A., & Tokgoz, S. (2008). Removing Distortions in the U.S. Ethanol Market: What Does It Imply for the United States and Brazil? *American Journal of Agricultural Economics*, *90*(4), 918–932. <https://doi.org/10.1111/j.1467-8276.2008.01158.x>
16. Elobeid, A., Tokgoz, S., Hayes, D. J., Babcock, B., & Hart, C. E. (2007). The long-run impact of corn-based ethanol on the grain, oilseed, and livestock sectors with implications for biotech crops. *AgBioForum*, *10*(1), 11–18. Scopus.
17. Fabiosa, J. F., Beghin, J. C., Dong, F., Elobeid, A., Tokgoz, S., & Yu, T.-H. (2010). The Global Bioenergy Expansion: How Large Are the Food−Fuel Trade-Offs? In M. Khanna, J. Scheffran, & D. Zilberman (Eds.), *Handbook of Bioenergy Economics and Policy* (pp. 113–132). Springer New York. <https://doi.org/10.1007/978-1-4419-0369-3_8>
18. Gohin, A. (2014). Assessing the Land Use Changes and Greenhouse Gas Emissions of Biofuels: Elucidating the Crop Yield Effects. *Land Economics*, *90*(4), 575–586.
19. Gohin, A., & Tréguer, D. (2010). On the (De)Stabilization Effects of Biofuels: Relative Contributions of Policy Instruments and Market Forces. *Journal of Agricultural and Resource Economics*, *35*(1). <http://econpapers.repec.org/article/agsjlaare/61061.htm>
20. Herreros, Ó. P., Peña, R. P., & Sánchez, J. E. S. (2014). *Economic Impact of the US EPA’s 2013 Renewable Fuel Standard on International Corn Prices and Mexican Welfare*. <https://doi.org/10.13140/2.1.2943.4249>
21. Jones, J. P. H., & McCarl, B. A. (2016). *Impacts of U.S. Production-Dependent Ethanol Policy on Agricultural Markets* (No. 236258). Article 236258. Agricultural & Applied Economics Association Annual Meeting, Boston, MA. [https://ideas.repec.org//p/ags/aaea16/236258.html](https://ideas.repec.org/p/ags/aaea16/236258.html)
22. Jones, J., Wang, Z., McCarl, B., & Wang, M. (2017). Policy Uncertainty and the US Ethanol Industry. *Sustainability*, *9*(11), 2056. <https://doi.org/10.3390/su9112056>
23. McPhail, L. L., & Babcock, B. (2008a). *Ethanol, mandates, and drought: Insights from a stochastic equilibrium model of the US corn market* (CARD Working Papers). Iowa State University. <https://works.bepress.com/bruce-babcock/95/>
24. McPhail, L. L., & Babcock, B. (2008b). *Short-run price and welfare impacts of federal ethanol policies* (CARD Working Papers). Iowa State University. <https://works.bepress.com/bruce-babcock/196/>
25. Meyer, S., & Thompson, W. (2012). How Do Biofuel Use Mandates Cause Uncertainty? United States Environmental Protection Agency Cellulosic Waiver Options. *Applied Economic Perspectives and Policy*, *34*(4), 570–586. <https://doi.org/10.1093/aepp/pps033>
26. Park, H., & Fortenbery, T. R. (2007). The effect of ethanol production on the US national corn price. *NCCC-134*. Conference on Applied Commodity Price Analysis, Forecasting and Market Risk Management., Chicago, IL. <http://ageconsearch.tind.io/record/37565/files/confp10-07.pdf>
27. Pérez Peña, R., & Peláez Herreros, O. (2019). Maíz, etanol y bienestar en el marco de las políticas (inter)nacionales. *Agricultura Sociedad y Desarrollo*, *16*(1), 1–18. <https://doi.org/10.22231/asyd.v1i1.978>
28. Roberts, M. J., & Schlenker, W. (2013). Identifying Supply and Demand Elasticities of Agricultural Commodities: Implications for the US Ethanol Mandate. *American Economic Review*, *103*(6), 2265–2295. <https://doi.org/10.1257/aer.103.6.2265>
29. Roberts, M. J., & Tran, A. N. (2013). Conditional Suspension of the US Ethanol Mandate using Threshold Price inside a Competitive Storage Model. *2013 Annual Meeting, Washington, DC, Agricultural and Applied Economics Association*. <http://ageconsearch.umn.edu/bitstream/150717/2/AAEA2013_Tran.pdf>
30. Schmitz, A., Moss, C. B., & Schmitz, T. G. (2020). The Economic Effects of COVID-19 on the Producers of Ethanol, Corn, Gasoline, and Oil. *Journal of Agricultural & Food Industrial Organization*, *18*(2), 20200025. <https://doi.org/10.1515/jafio-2020-0025>
31. Searchinger, T., Heimlich, R., Houghton, R. A., Dong, F., Elobeid, A., Fabiosa, J., Tokgoz, S., Hayes, D., & Yu, T.-H. (2008). Use of U.S. Croplands for Biofuels Increases Greenhouse Gases Through Emissions from Land-Use Change. *Science*, *319*(5867), 1238–1240. <https://doi.org/10.1126/science.1151861>
32. Taheripour, F., Baumes, H., & Tyner, W. E. (2022). Economic Impacts of the U.S. Renewable Fuel Standard: An Ex-Post Evaluation. *Frontiers in Energy Research*, *10*. <https://www.frontiersin.org/articles/10.3389/fenrg.2022.749738>
33. Thompson, W., Meyer, S., & Westhoff, P. (2009). How does petroleum price and corn yield volatility affect ethanol markets with and without an ethanol use mandate? *Energy Policy*, *37*(2), 745–749.
34. Thompson, W., Whistance, J., Westhoff, P., & Binfield, J. (2012). *Renewable Fuel Standard Waiver Options during the Drought of 2012* (FAPRI-MU No. 11–12). Food and Agricultural Policy Research Institute at the University of Missouri.
35. Tyner, W. E., Ackerman, L., & Taheripour, F. (2012). *Potential Impacts of a Partial Waiver of the Ethanol Blending Rules*. Farm Foundation. <http://citeseerx.ist.psu.edu/viewdoc/download?doi=10.1.1.368.2986&rep=rep1&type=pdf>
36. Tyner, W. E., & Taheripour, F. (2008). Policy options for integrated energy and agricultural markets. *Applied Economic Perspectives and Policy*, *30*(3), 387–396.
37. Wu, J., & Langpap, C. (2014). The Price and Welfare Effects of Biofuel Mandates and Subsidies. *Environmental and Resource Economics*, 1–23. <https://doi.org/10.1007/s10640-014-9814-8>
38. Zhou, W., & Babcock, B. A. (2017). Using the competitive storage model to estimate the impact of ethanol and fueling investment on corn prices. *Energy Economics*, *62*, 195–203. <https://doi.org/10.1016/j.eneco.2016.12.017>
